# Supplementary material for: A Comprehensive In Silico Study of New Metabolites from Heteroxenia fuscescens with SARS-CoV-2 Inhibitory Activity
Source: Molecules. 2022 Oct 29;27(21):7369. doi: 10.3390/molecules27217369 (PMC9657797; doi:10.3390/molecules27217369)
Supplement: Supplementary file 1 [file molecules-27-07369-s001.zip › molecules-1952247-supplementary.pdf]

Supplementary Material

A Comprehensive In Silico Study of New Metabolites from *Heteroxenia fuscescens* with SARS-CoV-2 Inhibitory Activity

Fahd M. Abdelkarem [1], Alaa M. Nafady [1], Ahmed E. Allam [1,*], Mahmoud A. H. Mostafa [1,2], Rwaida A. Al Haidari [2], Heba Ali Hassan [3], Magdi E. A. Zaki [4,*], Hamdy K. Assaf [1], Mohamed R. Kamel [1], Sabry A. H. Zidan [1], Ahmed M. Elsayed [5] and Kuniyoshi Shimizu [6]

[1] Department of Pharmacognosy, Faculty of Pharmacy, Al-Azhar University, Assiut 71524, Egypt
[2] Department of Pharmacognosy and Pharmaceutical Chemistry, College of Pharmacy, Taibah University, Al Madinah Al Munawarah 41477, Saudi Arabia
[3] Department of Pharmacognosy, Faculty of Pharmacy, Sohag University, Sohag 82524, Egypt
[4] Department of Chemistry, Faculty of Science, Imam Mohammad Ibn Saud Islamic University, Riyadh 13318, Saudi Arabia
[5] Department of Pharmacognosy, Faculty of Pharmacy, Nahda University, Beni-Suef 62513, Egypt
[6] Department of Agro-Environmental Sciences, Graduate School of Bioresource and Bioenvironmental Sciences, Kyushu University, Fukuoka 819-0395, Japan
* Correspondence: ahmedallam@azhar.edu.eg (A.E.A.); mezaki@imamu.edu.sa (M.E.A.Z.)

Content:

Table S1. ^{1}H NMR data of compounds **2, 3** and **5.**

Table S2. ^{1}H NMR data of compounds **6, 7** and **8.**

Table S3. ^{13}C-NMR data of compounds **2, 3, 5, 6, 7** and **8.**

Figure S1. Positive HRFAB-MS spectrum of compound **1**.

Figure S2. ^{1}H-NMR spectrum of compound **1** (600 MHz, $CDCl_3$).

Figure S3. Expanded ^{1}H-NMR spectrum (aromatic region) of compound **1** (600 MHz, $CDCl_3$).

Figure S4. ^{13}C-NMR spectrum of compound **1** (150 MHz, $CDCl_3$).

Figure S5. HSQC spectrum of compound **1** ($CDCl_3$).

Figure S6. HMBC spectrum of compound **1** ($CDCl_3$).

Figure S7. ^{1}H-^{1}H COSY spectrum of compound **1** ($CDCl_3$).

Figure S8. Expanded ^{1}H-^{1}H COSY spectrum of compound **1** ($CDCl_3$).

Figure S9. NOESY spectrum of compound **1** ($CDCl_3$).

Figure S10. Expanded NOESY spectrum of compound **1** ($CDCl_3$).

Figure S11. Positive HR-ESI-MS spectrum of compound **4**.

Figure S12. ^{1}H-NMR spectrum of compound **4** (600 MHz, CD_3OD).

Figure S13. ^{13}C-NMR spectrum of compound **4** (150 MHz, CD_3OD).

Figure S14. HSQC spectrum of compound **4** (CD_3OD).

Figure S15. HMBC spectrum of compound **4** (CD_3OD).

Figure S16. ^{1}H-^{1}H COSY spectrum of compound **4** (CD_3OD).

Figure S17. Expanded ^{1}H-^{1}H COSY spectrum of compound **4** (CD_3OD).

Figure S18. NOESY spectrum of compound **4** (CD_3OD).

Figure S19. Expanded NOESY spectrum of compound **4** (CD_3OD).

Figure S20. ^{1}H-NMR spectrum of compound **2** (600 MHz, $CDCl_3$).

Figure S21. ^{13}C-NMR spectrum of compound **2** (150 MHz, $CDCl_3$).

Figure S22. ^{1}H-NMR spectrum of compound **3** (600 MHz, $CDCl_3$).

Figure S23. ^{13}C-NMR spectrum of compound **3** (150 MHz, $CDCl_3$).

Figure S24. ^{1}H-NMR spectrum of compound **5** (600 MHz, CD_3OD).

Figure S25. ^{13}C-NMR spectrum of compound **5** (150 MHz, CD_3OD).

Figure S26. ^{1}H-NMR spectrum of compound **6** (600 MHz, CD_3OD).

Figure S27. ^{13}C-NMR spectrum of compound **6** (150 MHz, CD_3OD).

Figure S28. ^{1}H-NMR spectrum of compound **7** (600 MHz, CD_3OD).

Figure S29. ^{13}C-NMR spectrum of compound **7** (150 MHz, CD_3OD).

Figure S30. ^{1}H-NMR spectrum of compound **8** (600 MHz, CD_3OD).

Figure S31. ^{13}C-NMR spectrum of compound **8** (150 MHz, CD_3OD).

Table S4. All SARS CoV-2 related proteins used in this study.

Table S5. Docking scores of the isolated compounds (1–8) against the SARS CoV-2 targets expressed in kcal/mol.

Table S1. ^{1}H NMR data of compounds **2, 3** and **5.**

Position	2[a]			3[b]			5[c]		
	δH	Multip-licity	[J in (Hz)]	*δH*	Multip-licity	[J in (Hz)]	*δH*	Multip-licity	[J in (Hz)]
1	2.65	dd	13.1, 4.3	1.85	m	-	2.04	m	-
				1.06	m	-	1.76	m	-
2	2.09	ddd	16.6, 6.9,4.6	1.82	m	-	1.72	m	-
	1.74	m	-	1.48	m	-	1.49	m	-
3	4.23	br.d	7.6	3.52	m	-	4.00	m	-
4	-	-	-	2.30	m	-	2.05	m	-
				2.28	m	-	1.53	m	-
5	5.37	br.t	1.6	-	-	-	-	-	-
6	-	-	-	5.36	t	2.3	3.43	s	-
7	2.49	dd	5.7, 3.2	1.96	m	-	1.50	m	-
				1.56	m	-			
8	5.83	dd	9.7, 3.4	1.43	m	-	1.28	m	-
9	5.95	dd	9.7, 2.1	0.92	m	-	1.23	m	-
10	-	-	-	-	-	-	-	-	-
11	1.83	m	-	1.58	m	-	3.81	td	10.6, 4.8
12	1.00	d	7.0	2.02	m	-	2.27	dd	12.2, 4.9
				1.17	m	-	1.43	m	-
13	0.73	d	7.0	-	-	-	-	-	-
14	1.32	s	-	0.97	m	-	1.16	m	-
15	1.88	d	1.2	1.08	m	-	1.58	m	-
							1.08	m	-
16				1.98	m	-	2.08	m	-
				1.30	m	-	1.36	m	-
17				1.21	m	-	1.29	m	-
18				0.66	s	-	0.71	s	-
19				1.03	s	-	1.26	s	-

**20**				**1.00**	**m**	**-**	**1.03**	**m**	**-**
21				0.94	s	-	1.05	s	-
22				0.16	m	-	0.18	m	-
23				-	-	-	-	-	-
24				0.24	dq	9.0, 7.2	0.25	m	-
25				1.54	m	-	1.56	m	-
26				0.86	d	6.6	0.88	d	6.5
27				0.96	d	6.6	0.97	d	6.7
28				0.94	d	6.6	0.96	d	6.7
29				0.92	s	-	0.92	s	-
30				0.46<br>−0.13	dd<br>dd	9.1, 4.3<br>5.6, 4.6	0.48<br>−0.10	dd<br>dd	9.1, 4.3<br>5.3, 4.9

[a,b]Measured in $CDCl_3$, ^1H NMR (600 MHz), [c]Measured in CD_3OD, ^1H NMR (600 MHz).

Table S2. ^1H NMR data of compounds **6, 7** and **8.**

Position	6[a]			7[a]			8[a]		
	δH	Multip-licity	[J in (Hz)]	*δH*	Multip-licity	[J in (Hz)]	*δH*	Multip-licity	[J in (Hz)]
1	1.85<br>1.13	m<br>m	-<br>-	1.90<br>1.46	mm	-<br>-	1.73<br>1.23	mm	-<br>-
2	1.72<br>1.46	m<br>m	-<br>-	1.73<br>1.49	mm	-<br>-	1.95<br>1.49	mm	-<br>-
3	3.97	m	-	3.98	m	-	4.01	m	-
4	2.01<br>1.53	m<br>m	-<br>-	2.05<br>1.54	mm	-<br>-	2.05<br>1.58	mm	-<br>-
5	-	-	-	-	-	-	-	-	-
6	3.43	br.s	-	3.44	br.s	-	3.43	br.s	-
7	1.78<br>1.54	m<br>m	-<br>-	1.79<br>1.42	mm	-<br>-	1.82<br>1.63	mm	-<br>-
8	1.84	m	-	1.88	m	-	1.77	m	-
9	1.86	m	-	1.43	m	-	1.45	m	-
10	-	-	-	-	-	-	-	-	-
11	5.12	td	10.6, 4.8	3.86	td	10.6, 5.1	1.54<br>1.37	mm	-<br>-
12	2.27<br>1.17	m<br>m	-<br>-	2.28<br>1.52	ddm	12.0, 5.4<br>-	1.34<br>1.63	mm	-<br>-
13	-	-	-	-	-	-	-	-	-
14	1.21	m	-	-	-	-	1.38	m	-
15	1.62<br>1.09	m<br>m	-<br>-	3.38	s	-	1.56<br>1.93	mm	-<br>-
16	1.37<br>1.35	m<br>m	-<br>-	1.78	m	-	2.44<br>2.09	ddm	19.3, 8.6<br>-
17	1.33	m	-	1.38	m	-	-	-	-
18	0.73	s	-	1.03	s	-	0.88	s	-

**19**	**1.23**	**s**	**-**	**1.25**	**s**	**-**	**1.18**	**s**	**-**
20	1.04	m	-	-	-	-			
21	1.00	s	-	1.23	s	-			
22	0.19	m	-	1.70<br>1.45	mm	-<br>-			
23	-	-	-	2.07	m	-			
24	0.25	dq	9.0, 6.0	1.18	m	-			
25	1.56	m	-	1.42	m	-			
26	0.88	d	6.6	0.89	d	6.6			
27	0.96	d	6.6	0.92	d	6.6			
28	0.98	d	6.6	0.78	d	6.6			
29	0.92	s	-	0.83	d	6.9			
30	0.48<br>- 0.11	dd<br>dd	9.0, 6.0<br>6.0, 4.0						
1'	-	-	-						
2'	2.00	s	-						

[a]Measured in CD_3OD, ^1H NMR (600 MHz).

Table S3. ^{13}C-NMR data of compounds **2, 3, 5, 6, 7** and **8.**

Position	2[a]	3[b]	5[c]	6[c]	7[c]	8[c]
	δC	δC	δC	δC	δC	δC
1	49.7	37.3	35.2	35.1	35.1	33.0
2	29.9	31.7	32.1	32.1	32.1	31.7
3	69.9	71.8	68.3	67.9	68.2	68.3
4	144.2	42.3	42.0	41.9	41.9	41.5
5	120.6	140.8	77.5	77.2	77.4	76.8
6	84.5	121.7	76.8	76.5	76.6	76.3
7	53.6	31.8	35.4	35.5	35.3	34.2
8	129.3	32.0	30.4	30.7	28.4	31.4
9	134.5	50.2	53.1	49.0	53.7	46.9
10	81.9	36.5	41.01	40.9	41.3	39.6
11	28.1	24.6	69.7	73.0	69.2	21.6
12	18.6	39.0	53.0	47.7	46.8	33.6
13	22.5	42.8	44.8	44.5	44.3	49.1
14	15.7	56.7	56.6	56.0	74.6	52.5
15	18.8	24.6	25.5	25.4	60.1	22.8
16		28.2	29.7	29.7	28.0	36.8
17		58.0	59.6	59.3	46.7	224.2
18		11.9	13.6	13.1	18.3	14.3
19		19.4	17.6	17.4	17.5	17.3
20		35.3	36.7	36.5	75.6	
21		21.2	21.6	21.2	27.1	
22		32.1	33.4	33.4	48.8	
23		25.8	26.8	26.8	30.2	
24		50.8	52.2	52.3	46.8	
25		32.2	33.3	33.3	32.2	
26		21.7	22.0	22.2	21.8	

27		22.2	22.7	22.1	21.6	

**28**		**15.4**	**16.0**	**16.0**	**11.9**	
29		14.3	14.7	14.7	15.9	
30		21.3	22.3	22.2		
1'				22.0		
2'				172.6		

[a,b]Measured in $CDCl_3$, ^1H NMR (600 MHz), [c]Measured in CD_3OD, ^1H NMR (600 MHz).

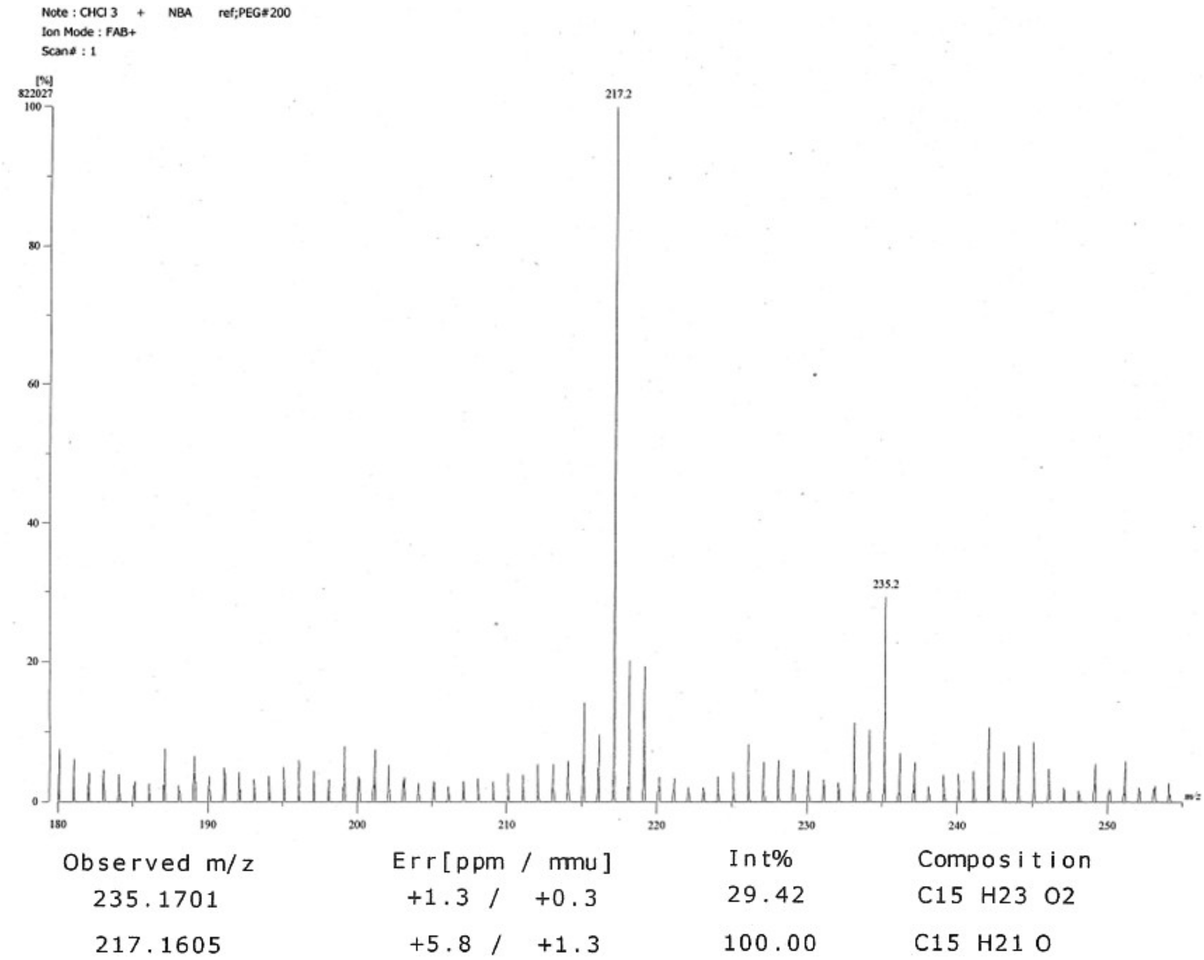

Figure S1. Positive HR-FAB-MS spectrum of compound **1**.

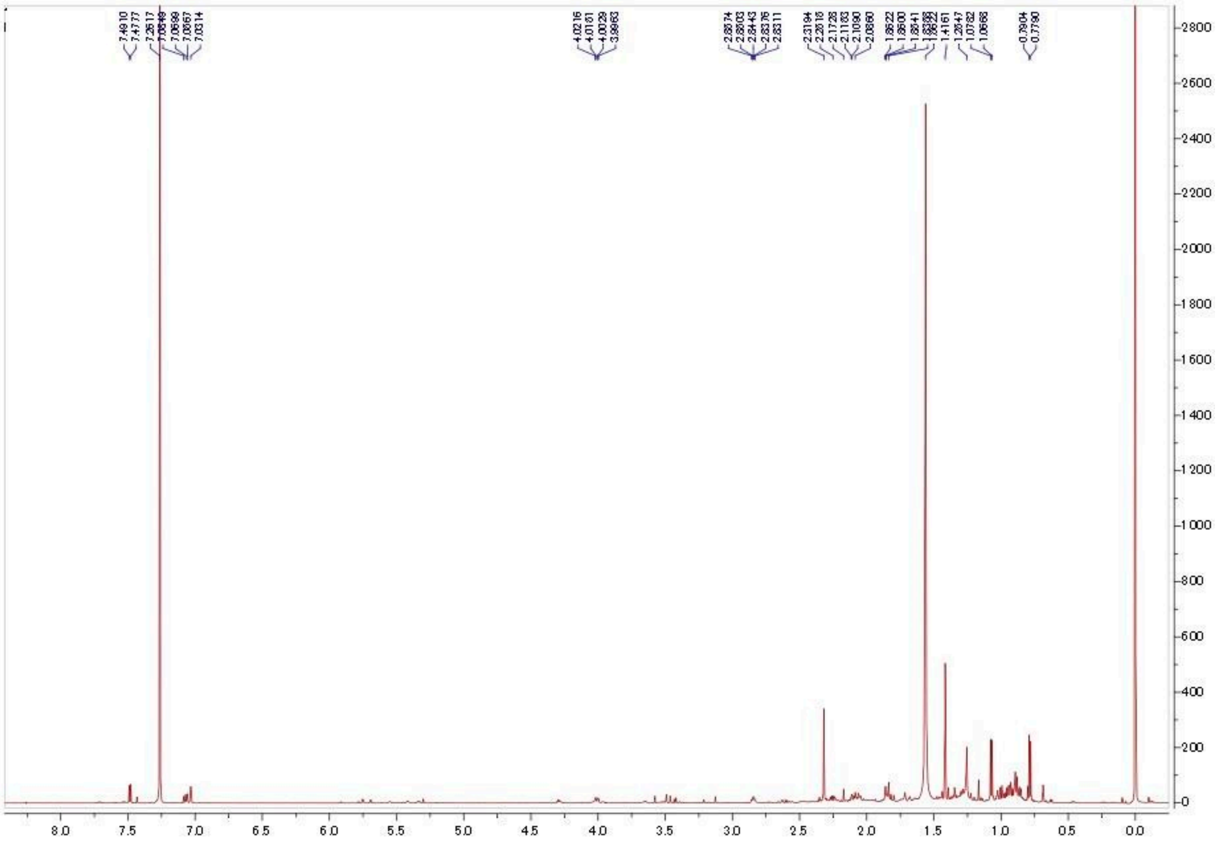

Figure S2. ^{1}H-NMR spectrum of compound **1** (600 MHz, $CDCl_3$).

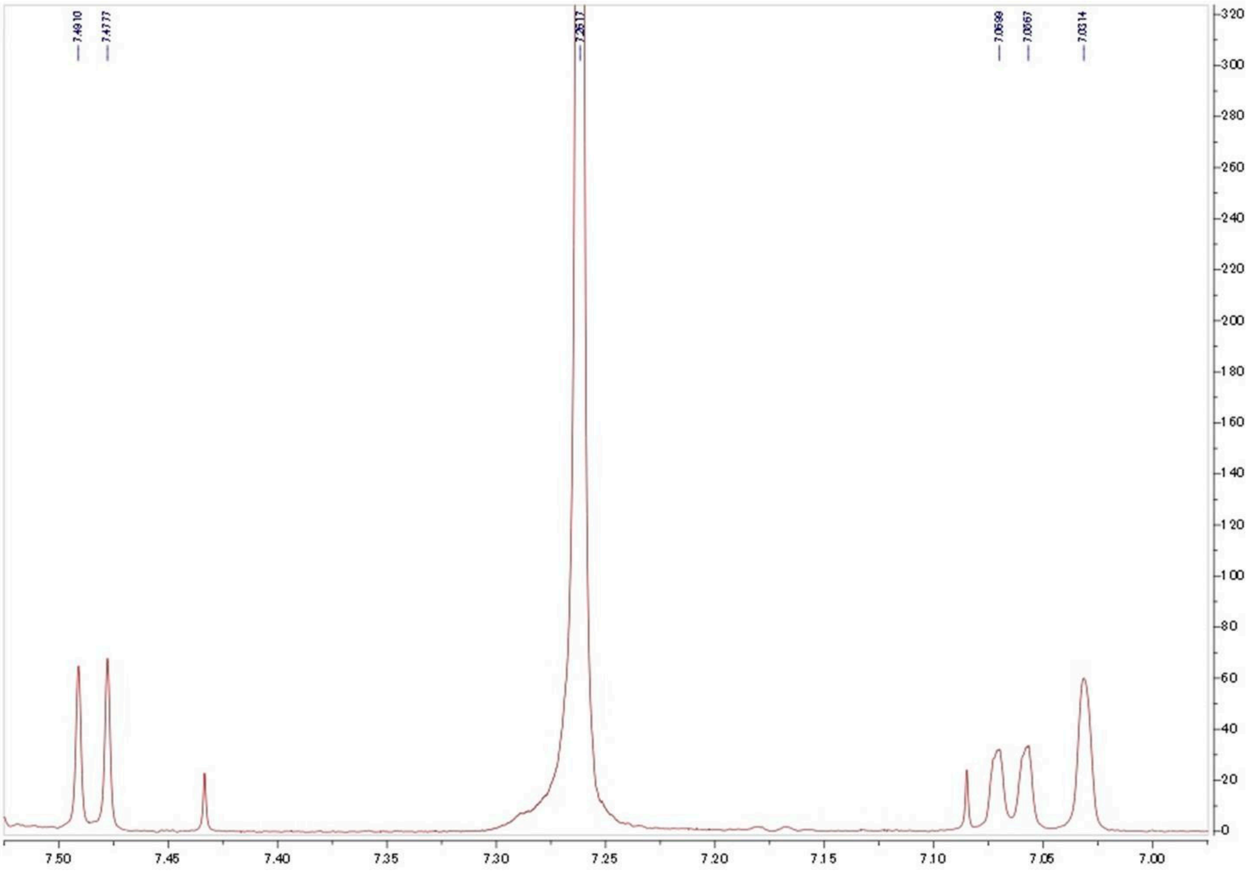

Figure S3. Expanded ^{1}H-NMR spectrum (aromatic region) of compound **1** (600 MHz, $CDCl_3$).

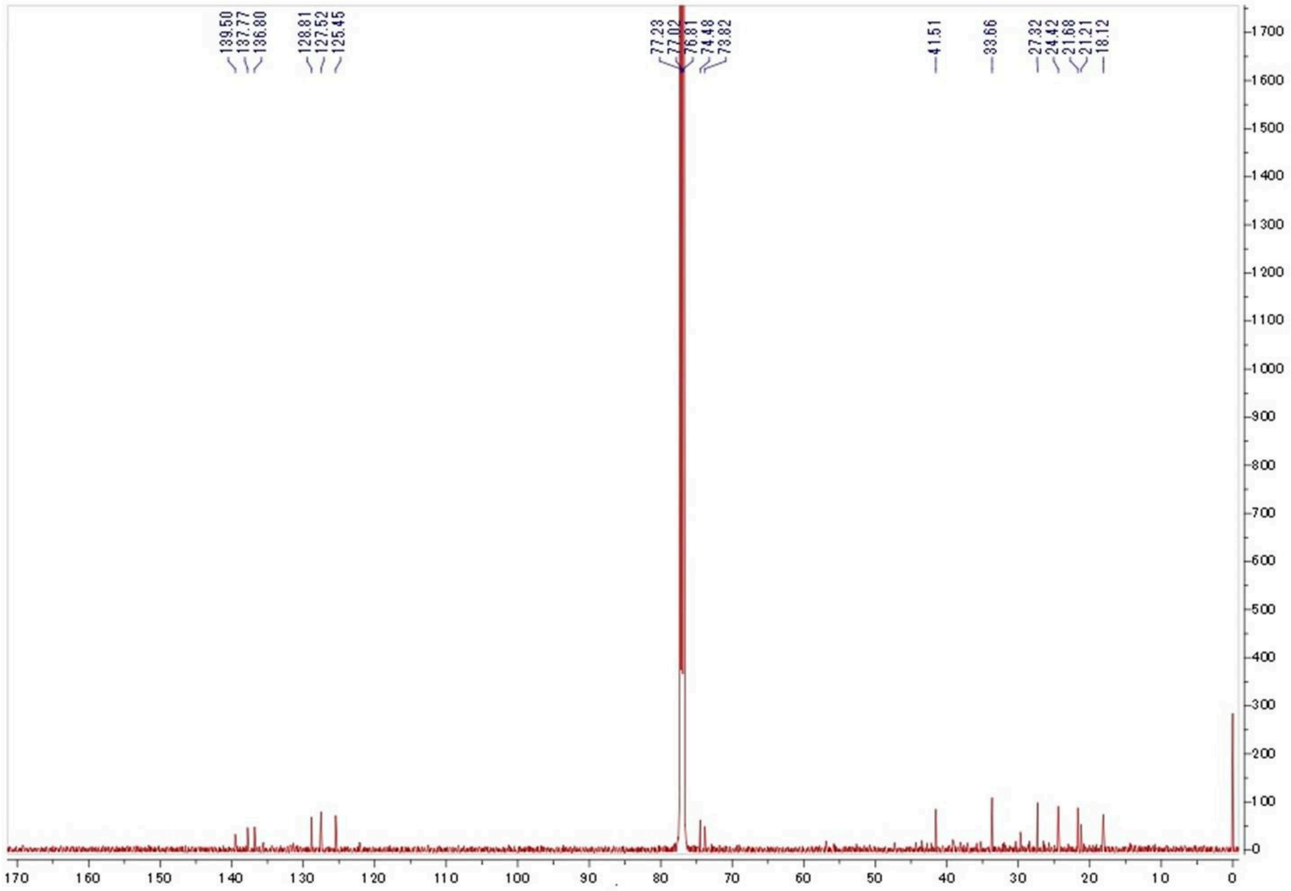

Figure S4. ^{13}C-NMR spectrum of compound **1** (150 MHz, $CDCl_3$).

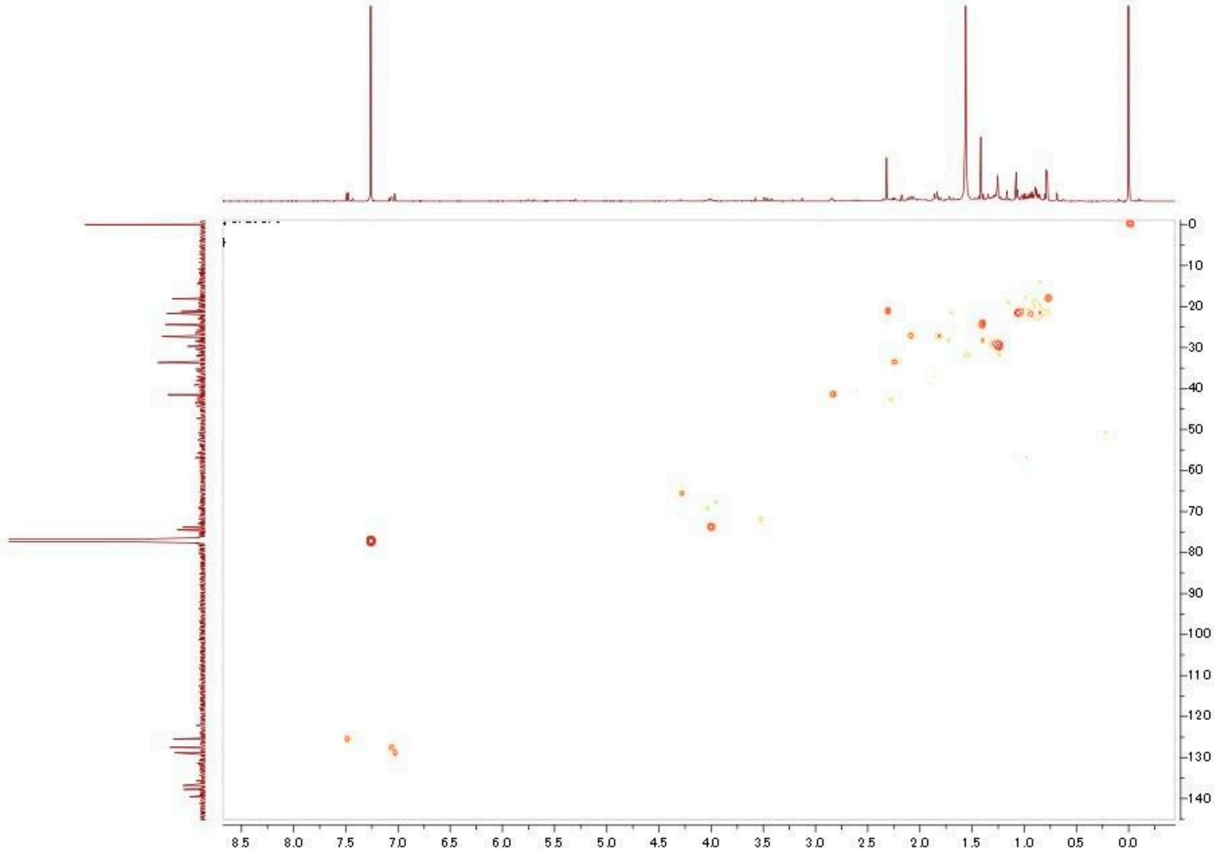

Figure S5. HSQC spectrum of compound **1** (CDCl3).

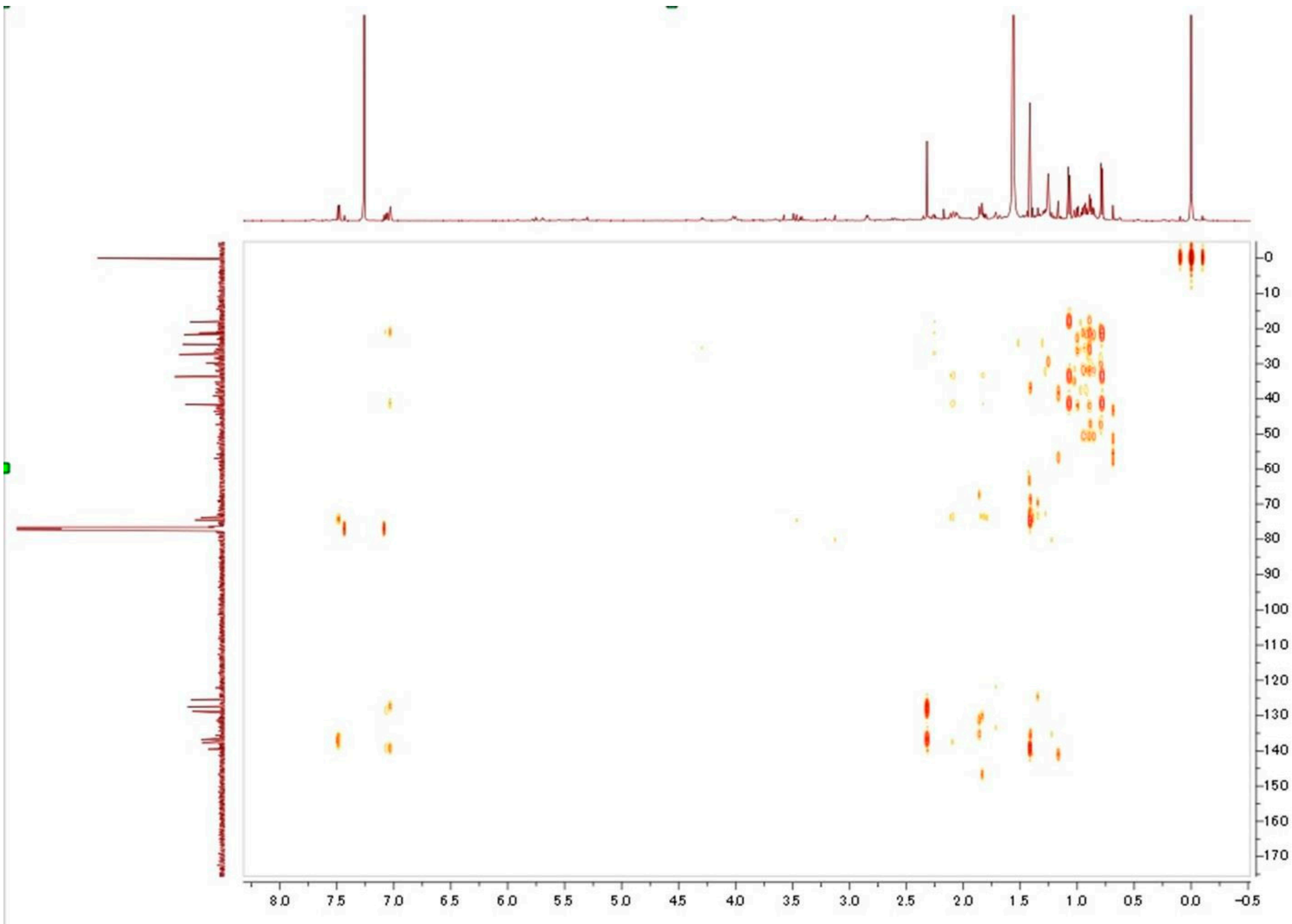

Figure S6. HMBC spectrum of compound **1** ($CDCl_3$).

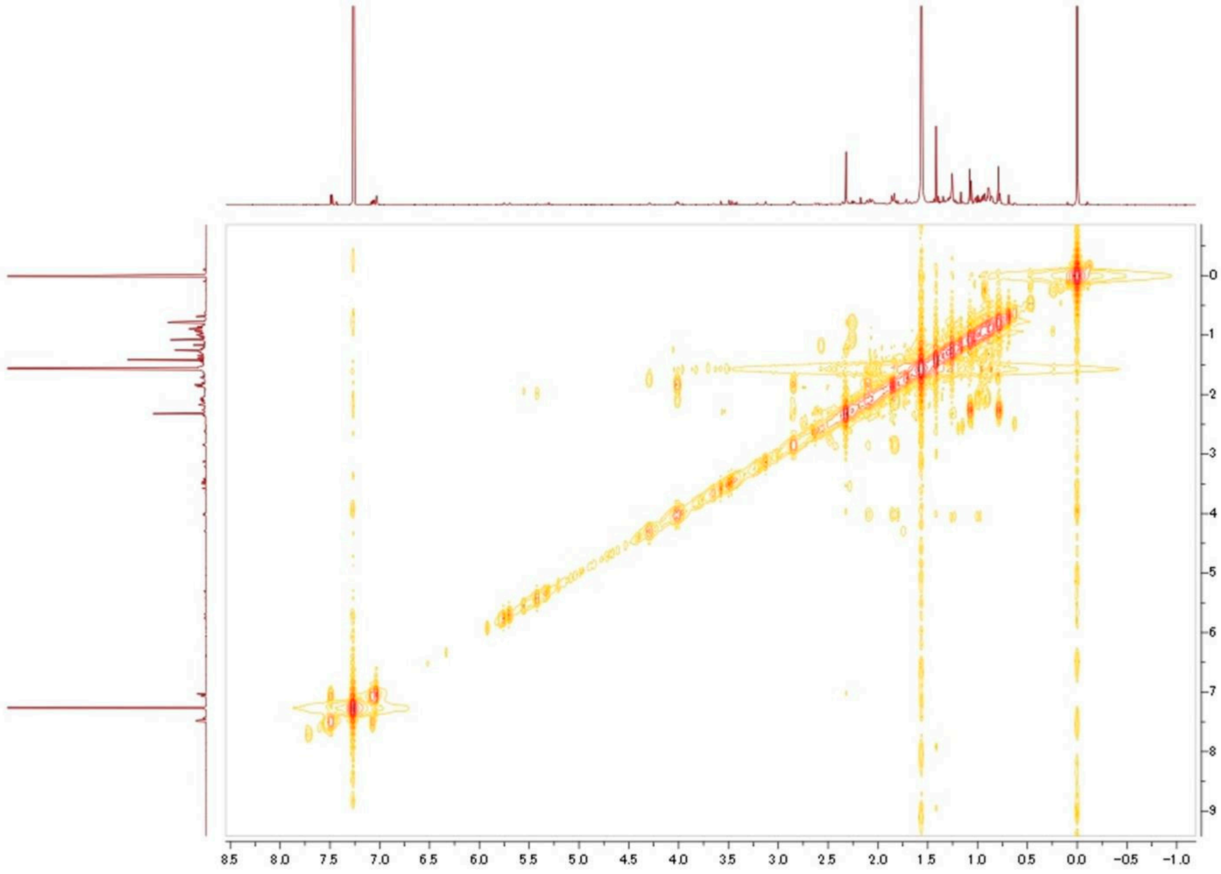

Figure S7. ^1H-^1H COSY spectrum of compound **1** ($CDCl_3$).

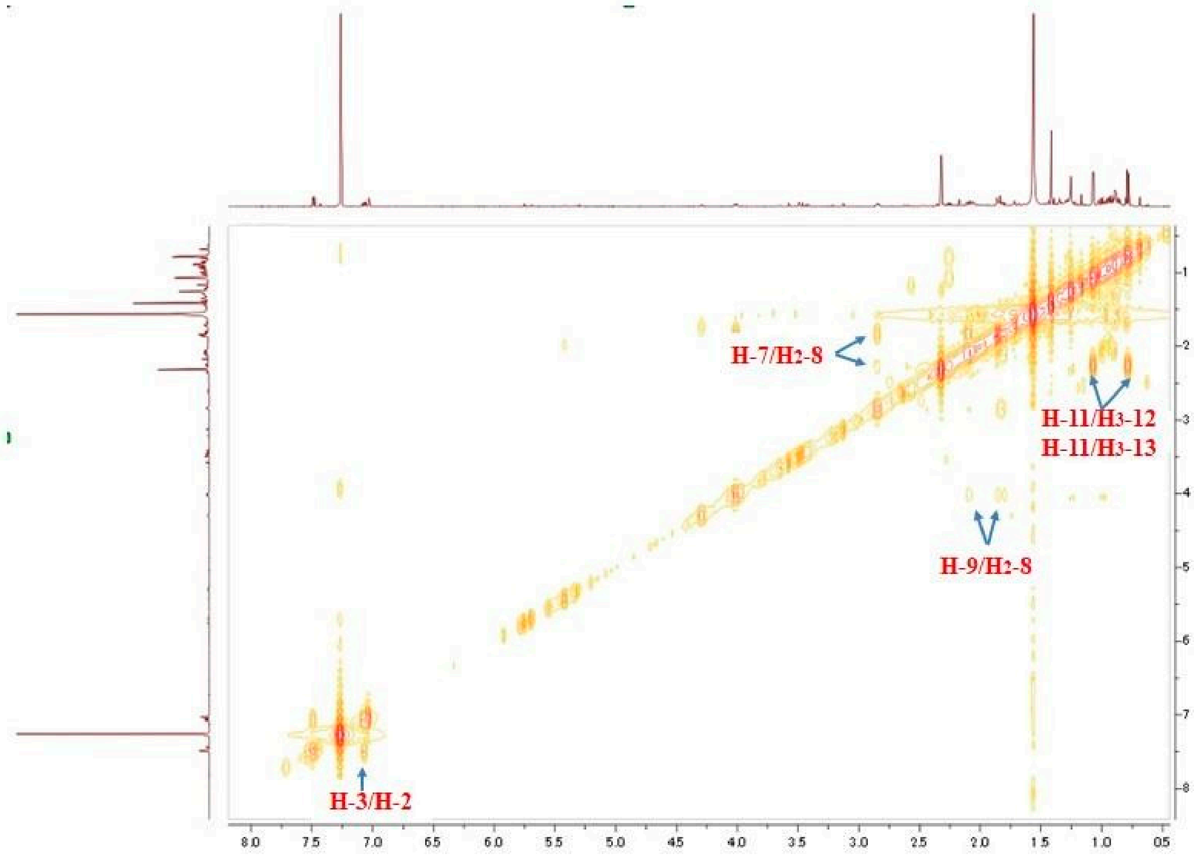

Figure S8. Expanded ^{1}H-^{1}H COSY spectrum of compound **1** ($CDCl_3$).

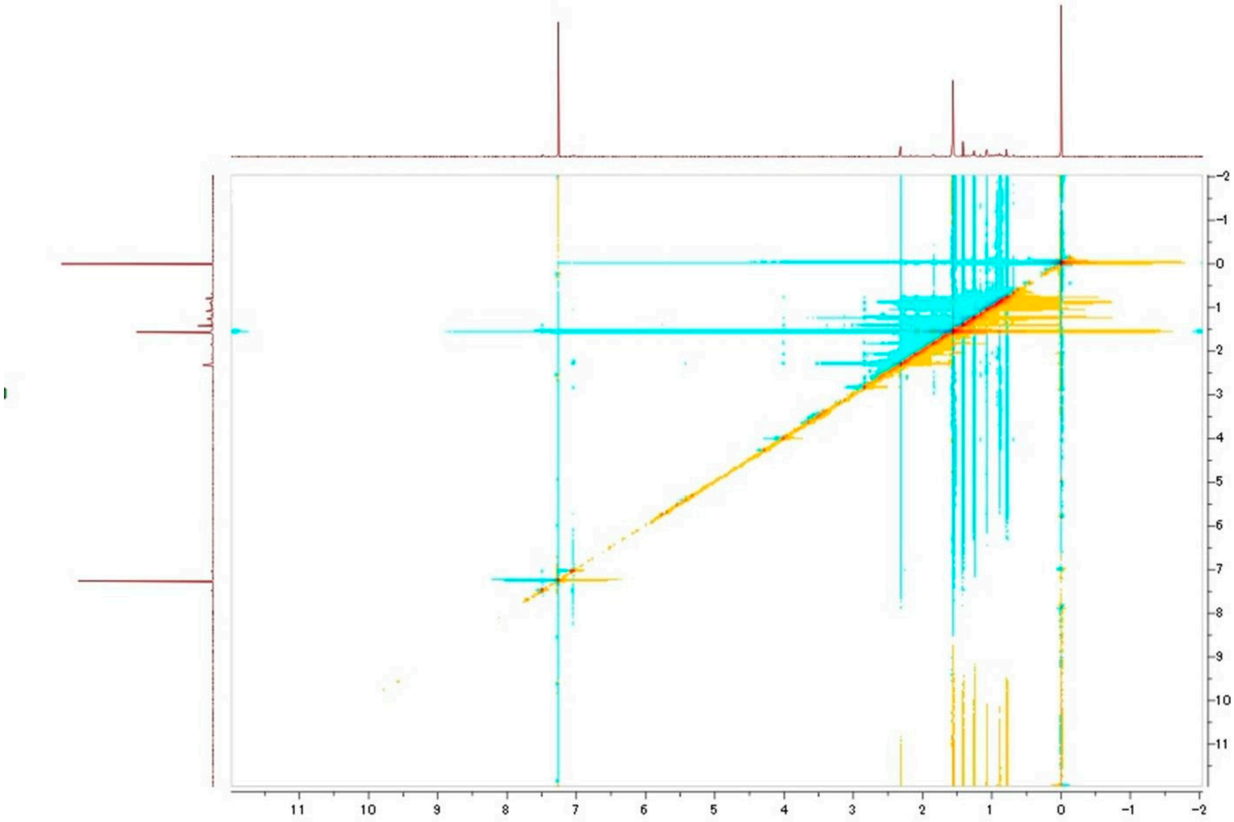

Figure S9. NOESY spectrum of compound **1** ($CDCl_3$).

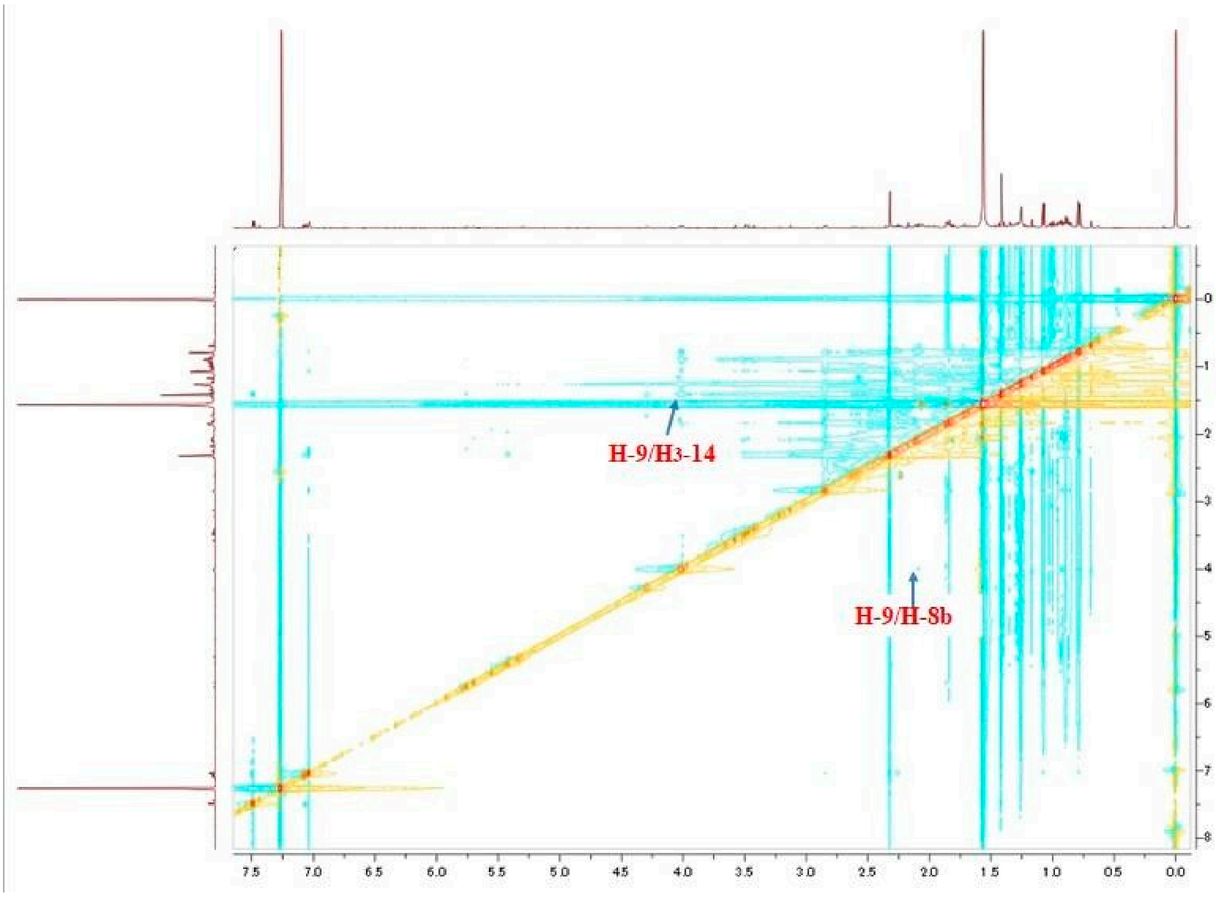

Figure S10. Expanded NOESY spectrum of compound **1** ($CDCl_3$).

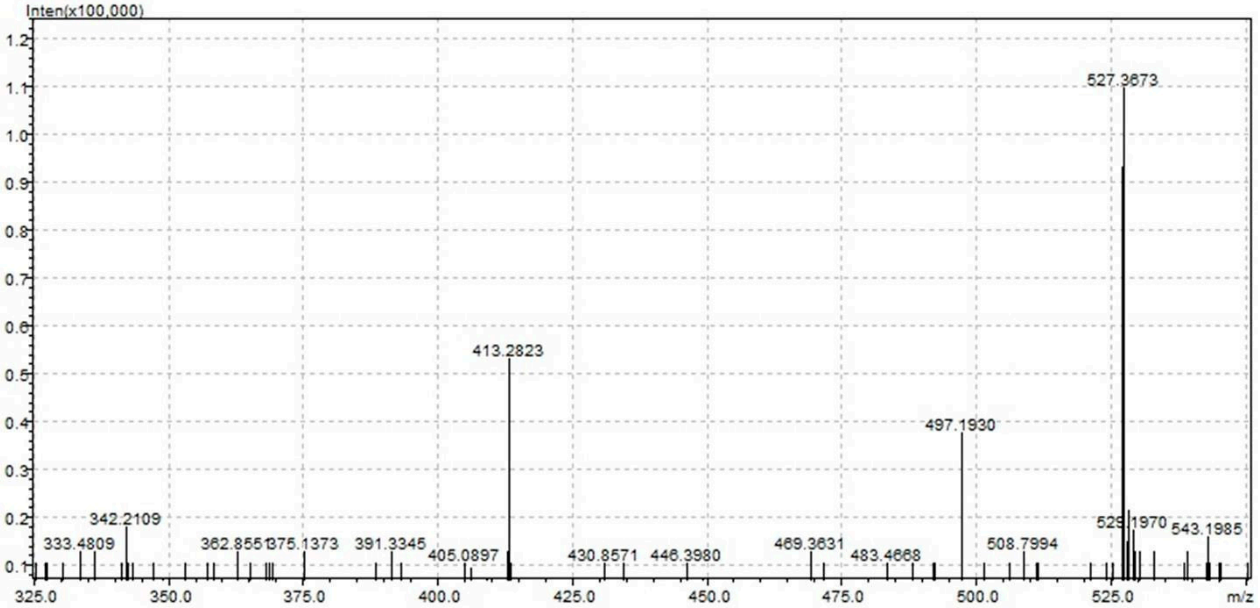

Molecular formula (M)	Ion	Meas. m/z	Pred. m/z	Diff (ppm)	DBE
$C_{31}H_{52}O_5$	[M+Na]+	527.3673	527.3712	-7.4	6.0

Figure S11. Positive HR-ESI-MS spectrum of compound **4**.

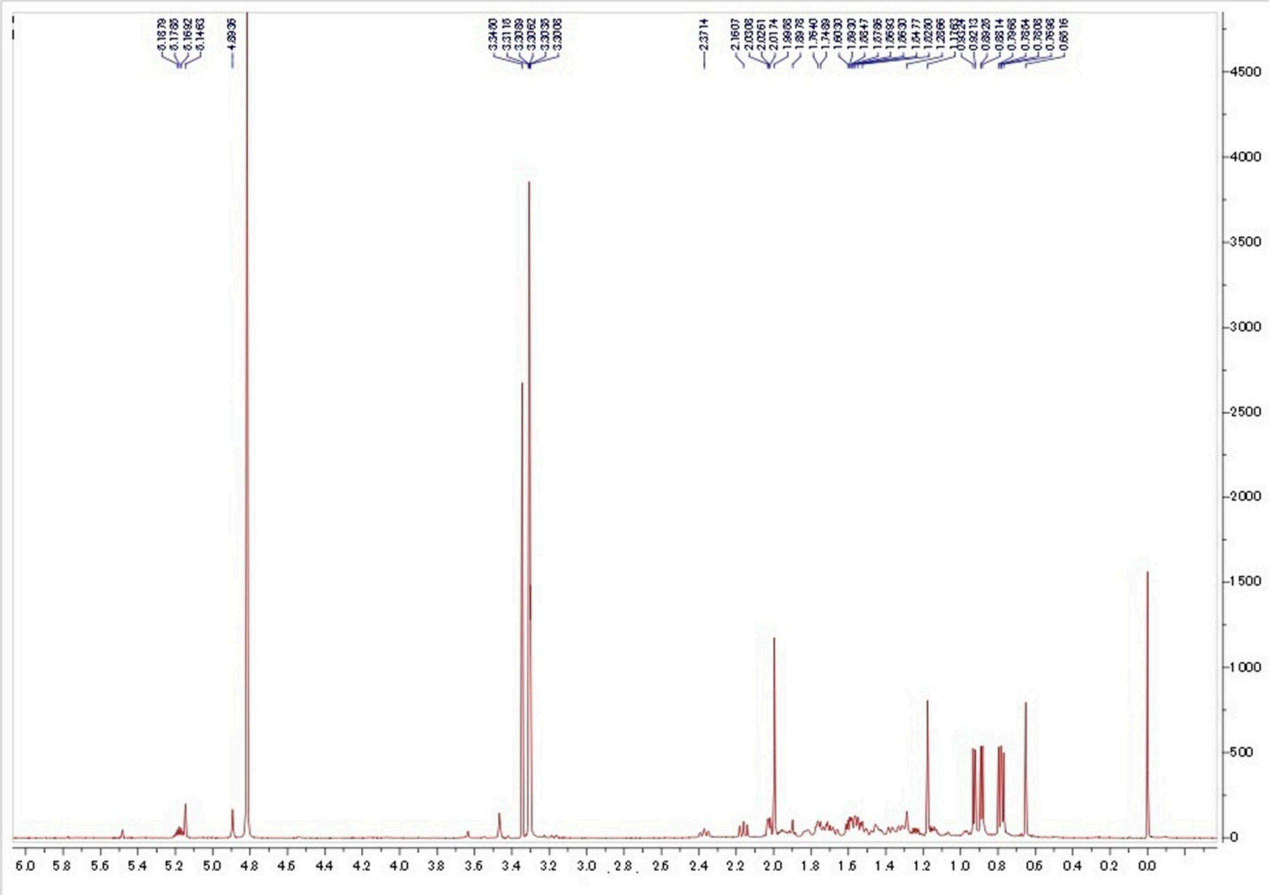

Figure S12. ^{1}H-NMR spectrum of compound **4** (600 MHz, CD_3OD).

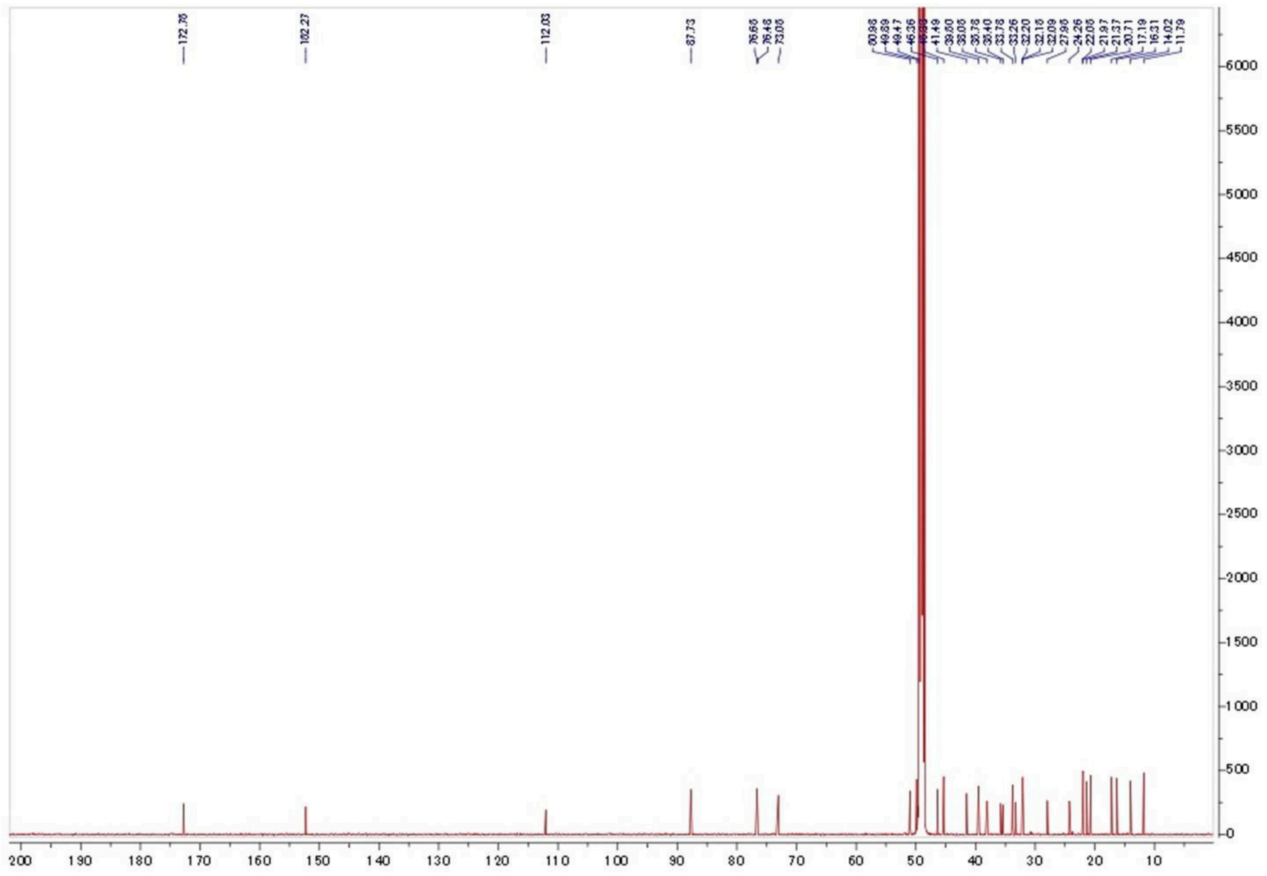

Figure S13. ^{13}C-NMR spectrum of compound **4** (150 MHz, CD3OD).

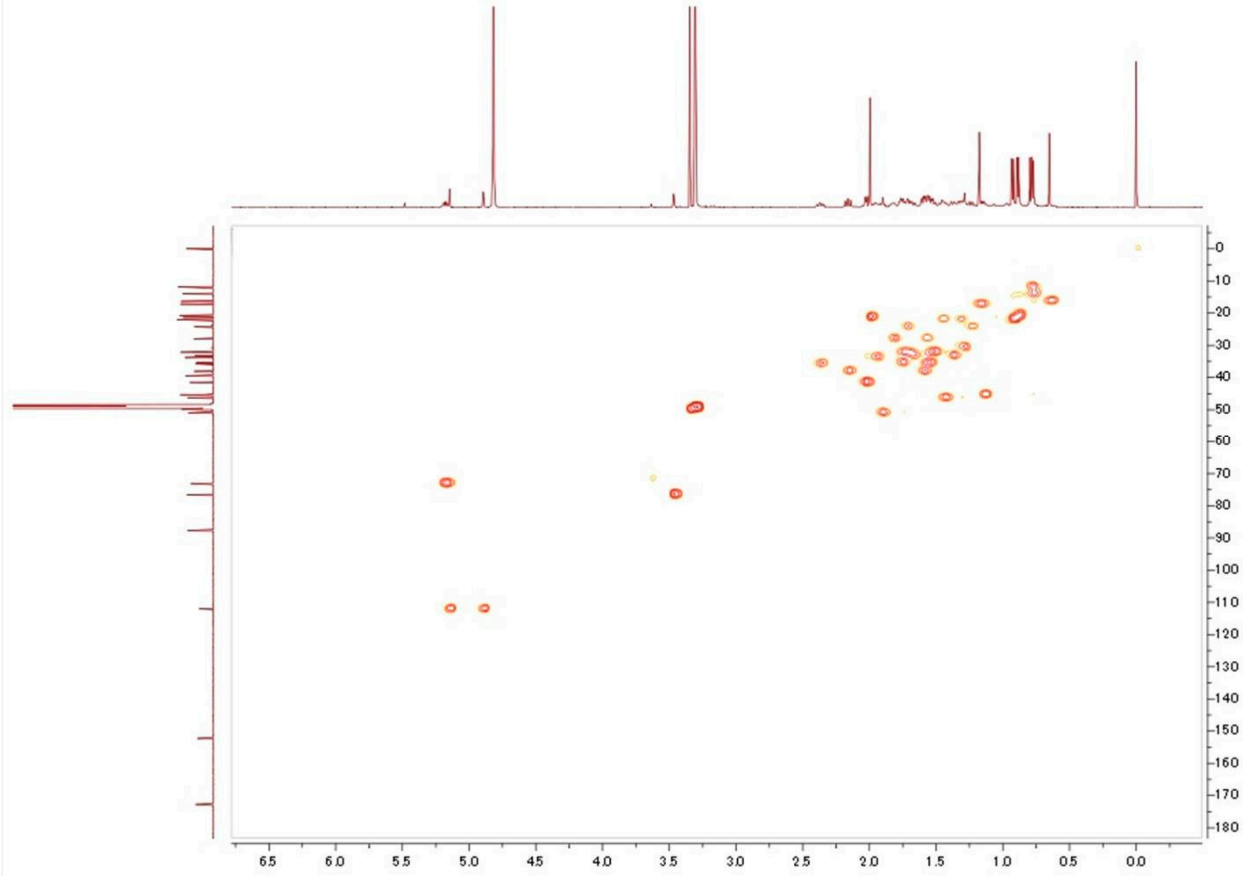

Figure S14. HSQC spectrum of compound **4** (CD_3OD).

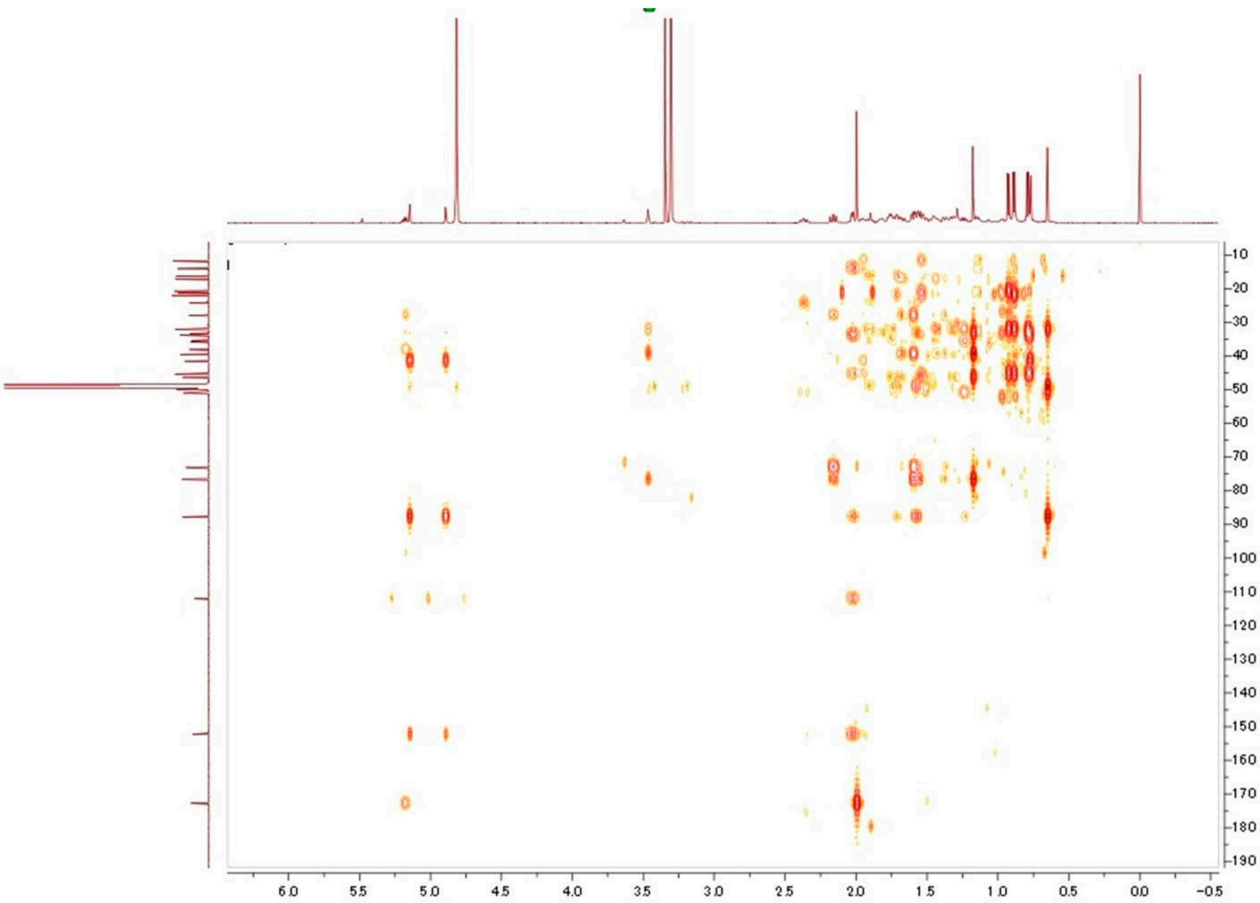

Figure S15. HMBC spectrum of compound **4** (CD3OD).

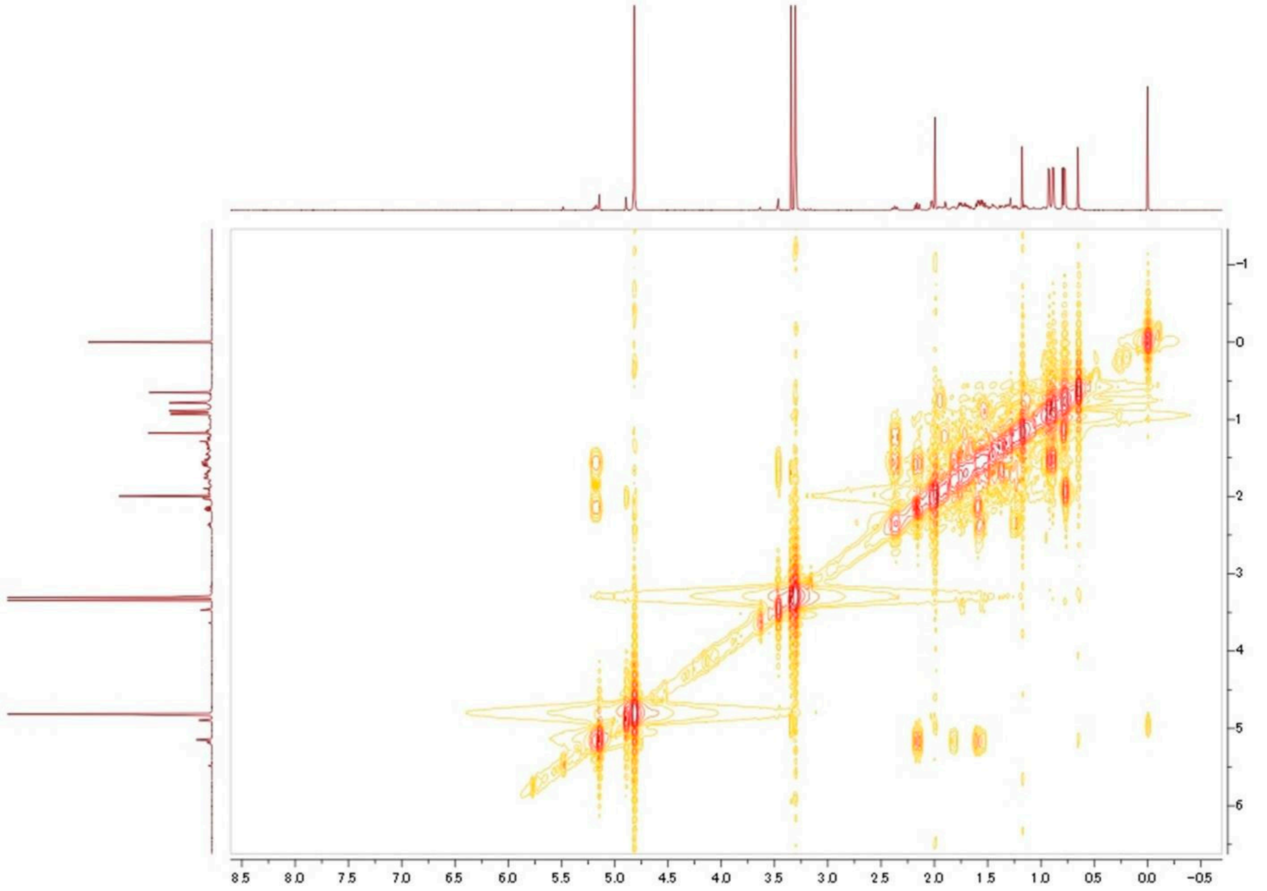

Figure S16. ^{1}H-^{1}H COSY spectrum of compound **4** (CD_3OD).

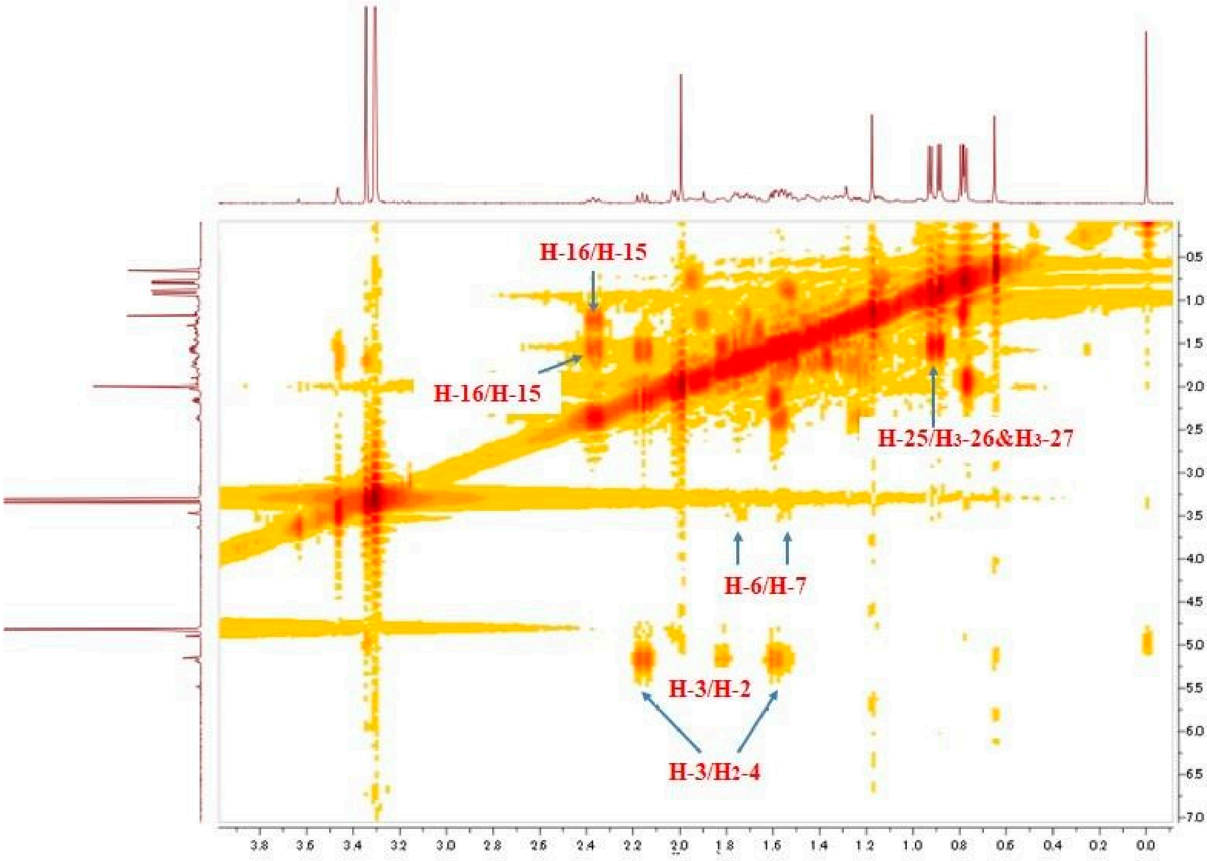

Figure S17. Expanded ^1H-^1H COSY spectrum of compound **4** (CD_3OD).

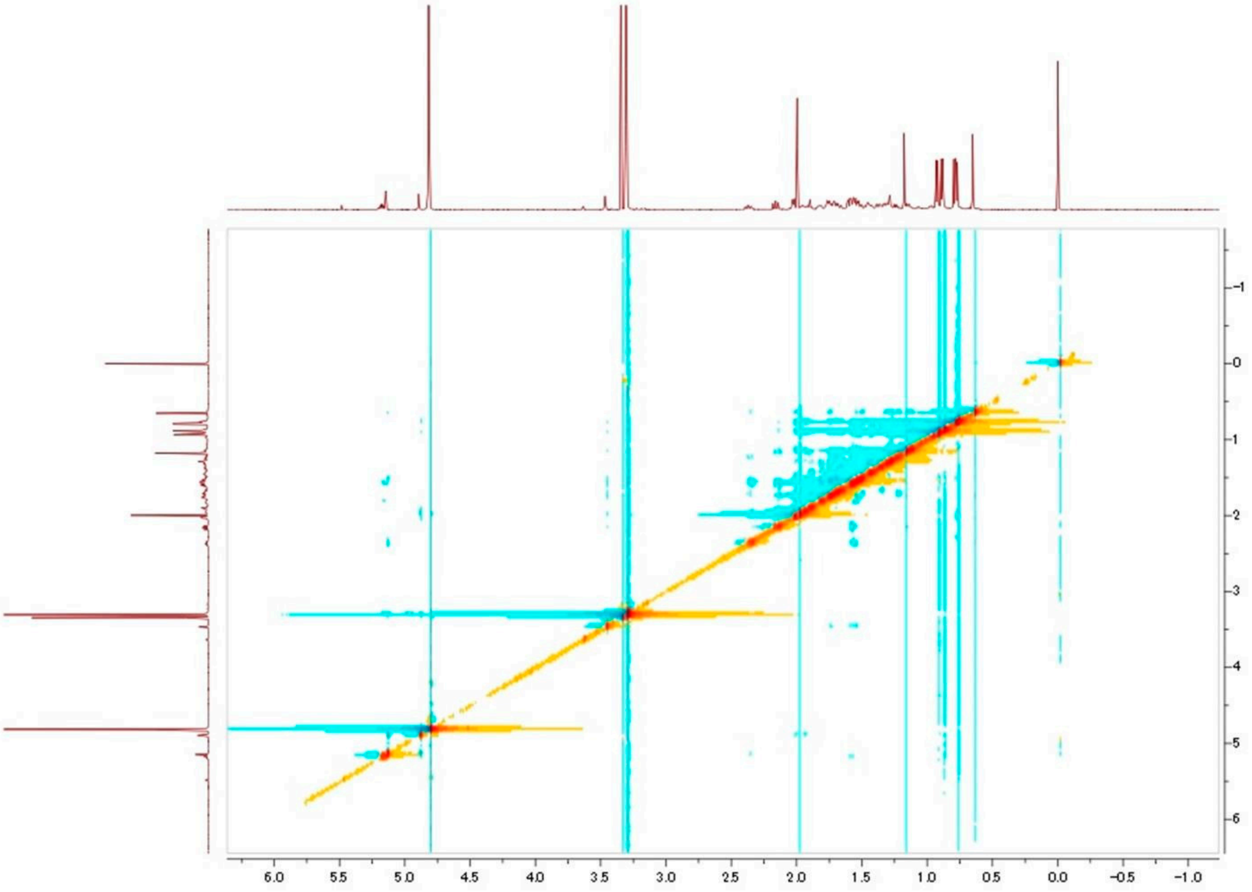

Figure S18. NOESY spectrum of compound **4** (CD3OD).

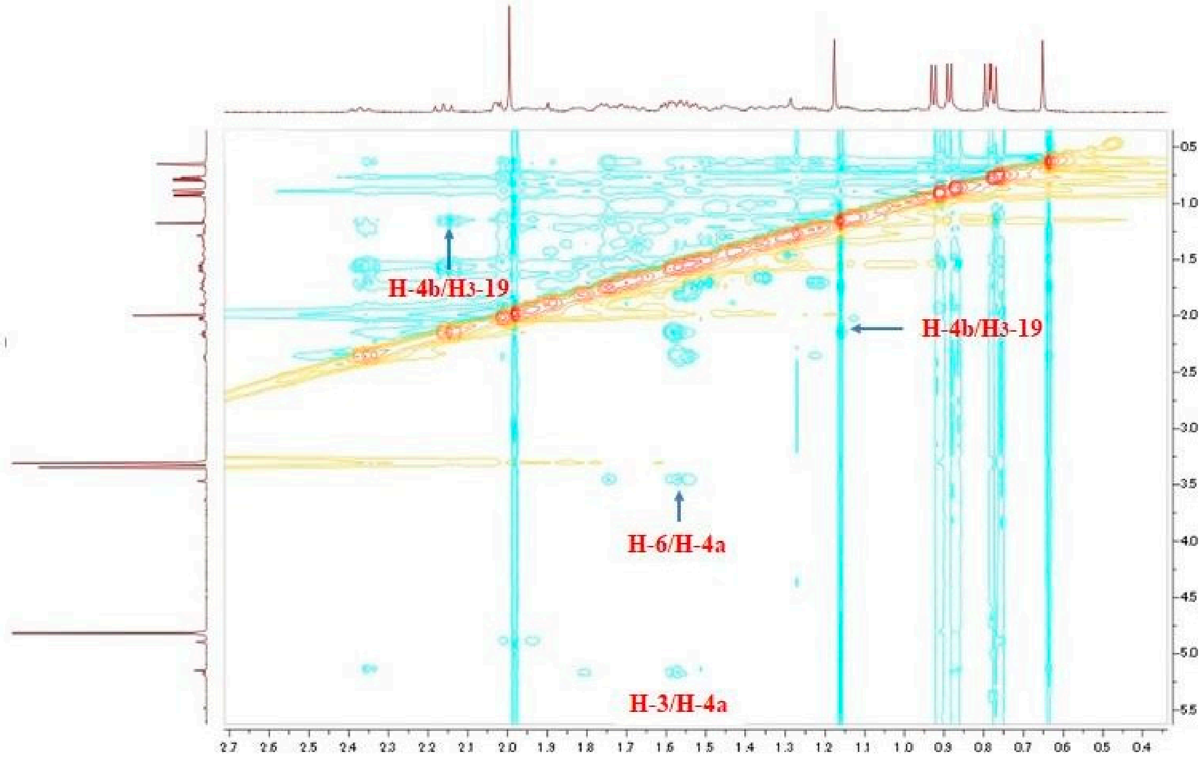

Figure S19. Expanded NOESY spectrum of compound **4** (CD_3OD).

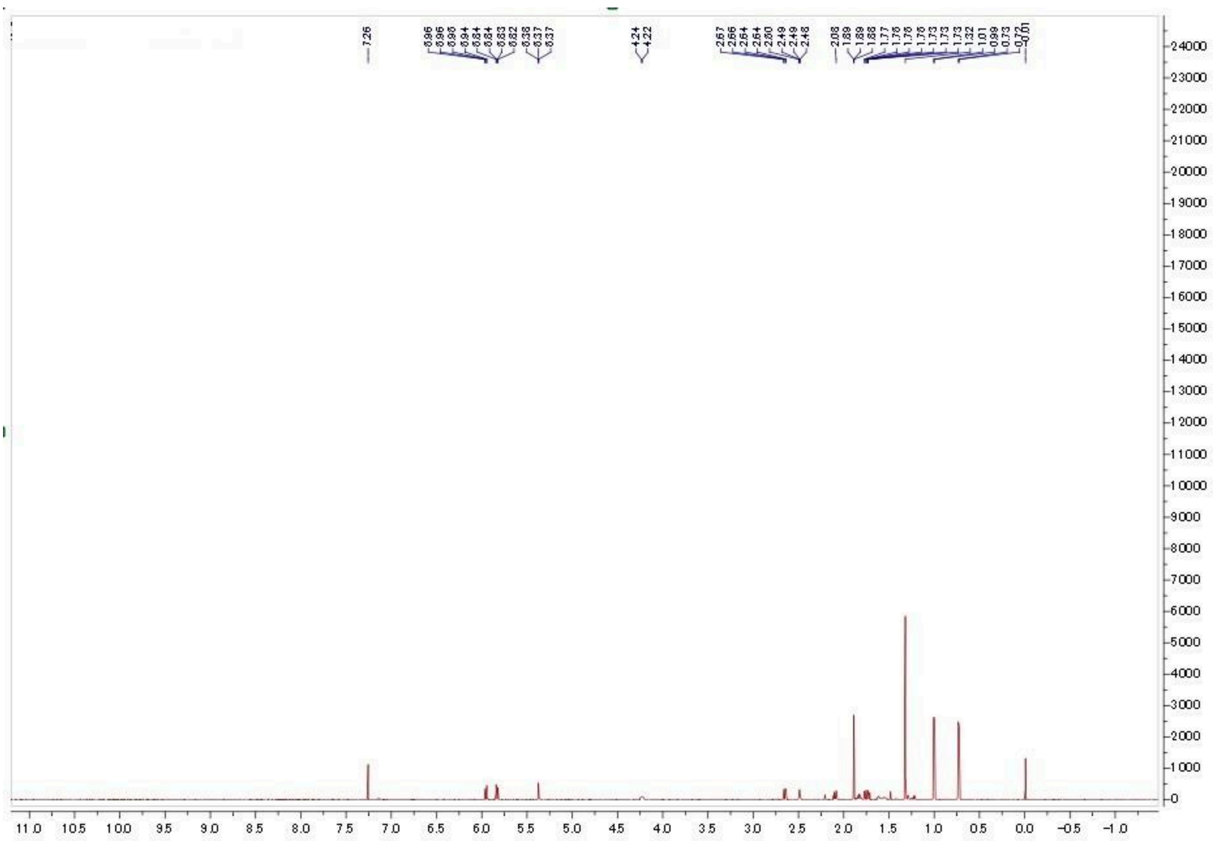

Figure S20. ^{1}H-NMR spectrum of compound **2** (600 MHz, $CDCl_3$).

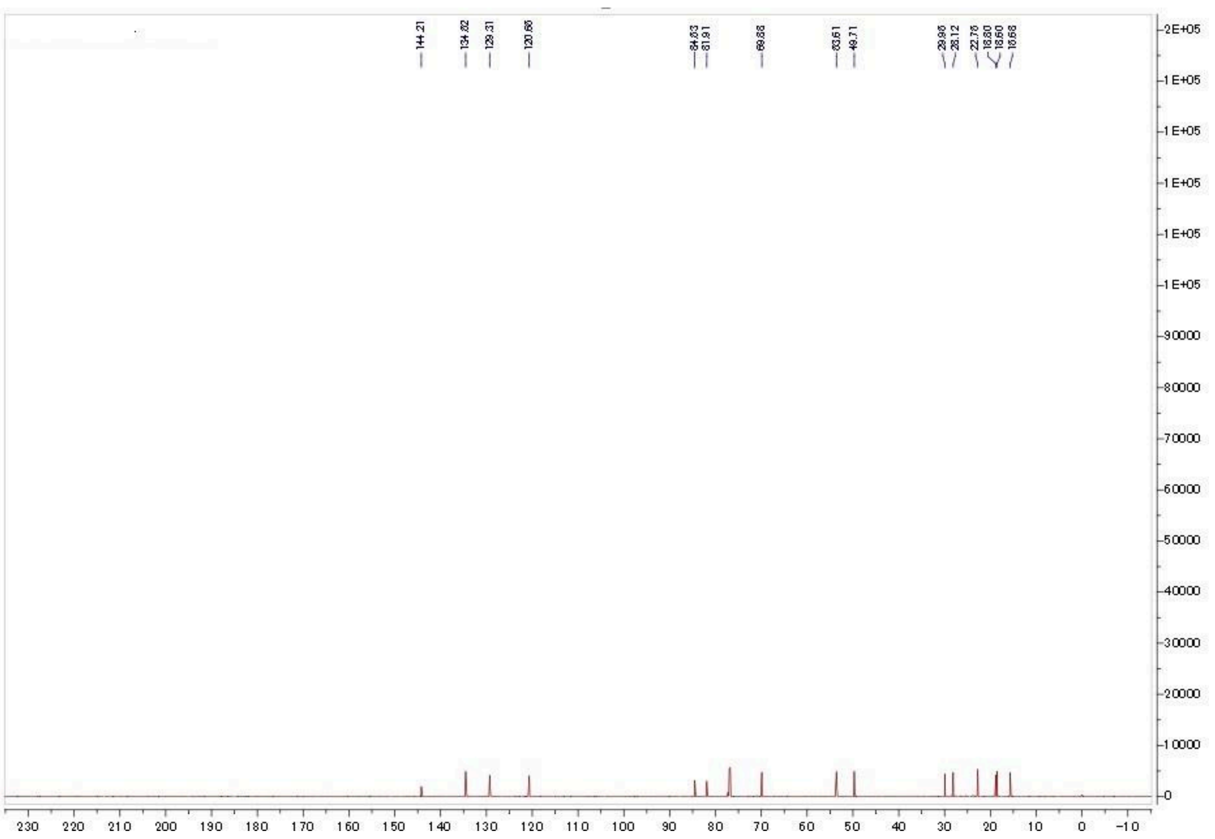

Figure S21. ^{13}C-NMR spectrum of compound **2** (150 MHz, $CDCl_3$).

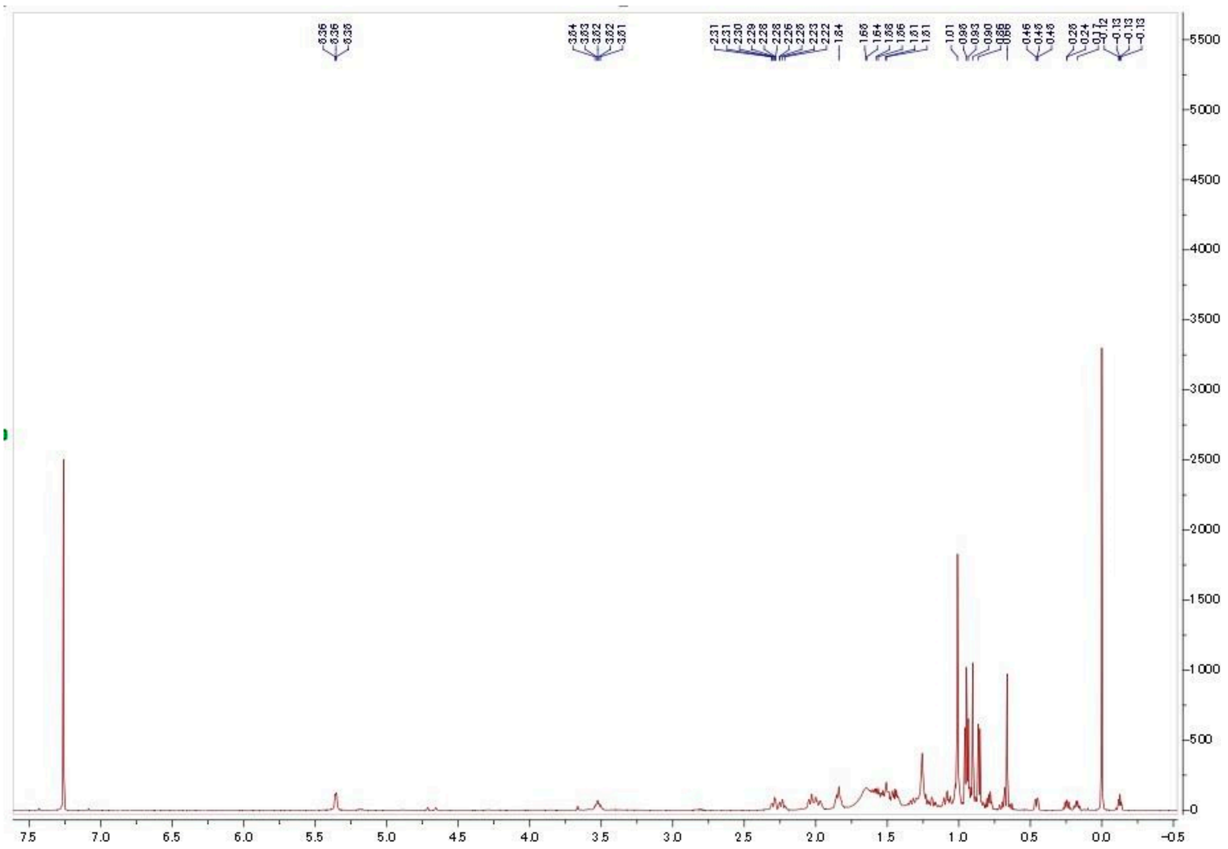

Figure S22. ^{1}H-NMR spectrum of compound **3** (600 MHz, $CDCl_3$).

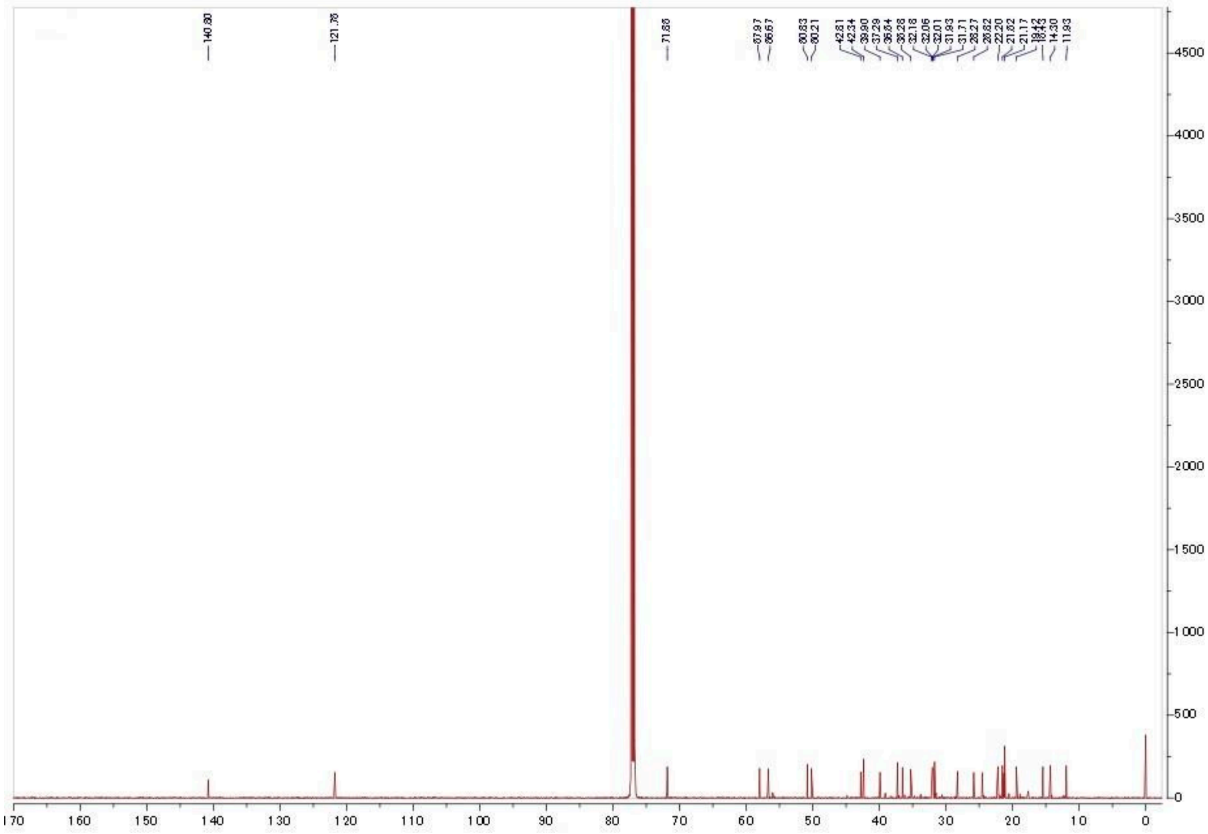

Figure S23. ^{13}C-NMR spectrum of compound **3** (150 MHz, $CDCl_3$).

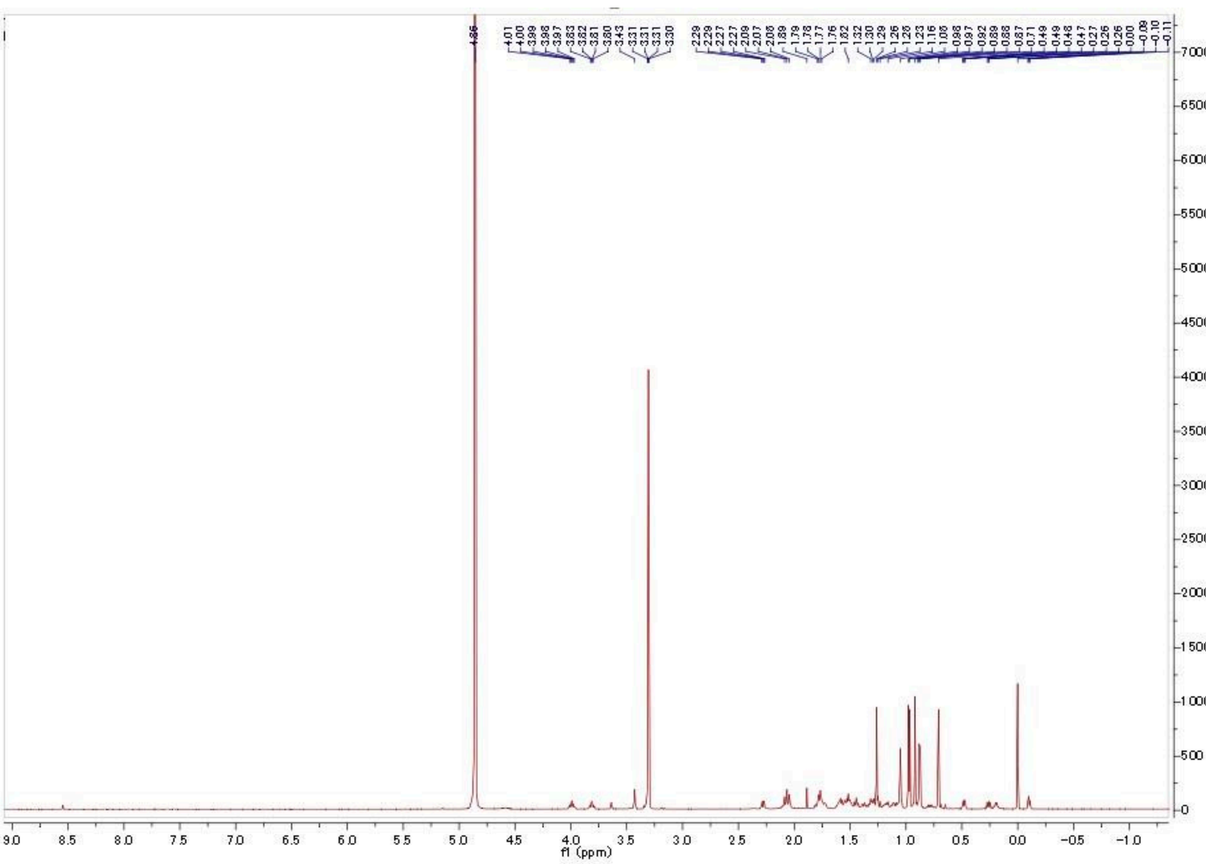

Figure S24. ^{1}H-NMR spectrum of compound **5** (600 MHz, CD3OD).

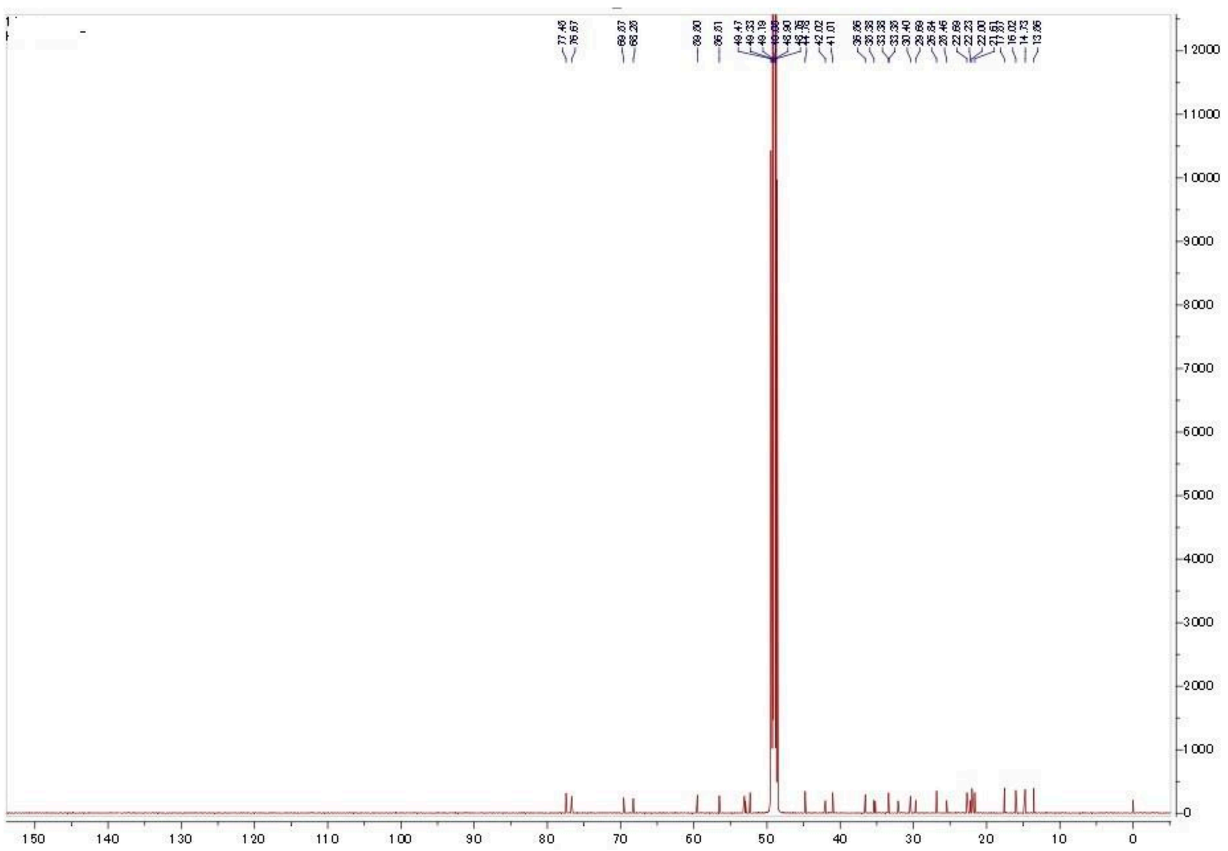

Figure S25. ^{13}C-NMR spectrum of compound **5** (150 MHz, CD3OD).

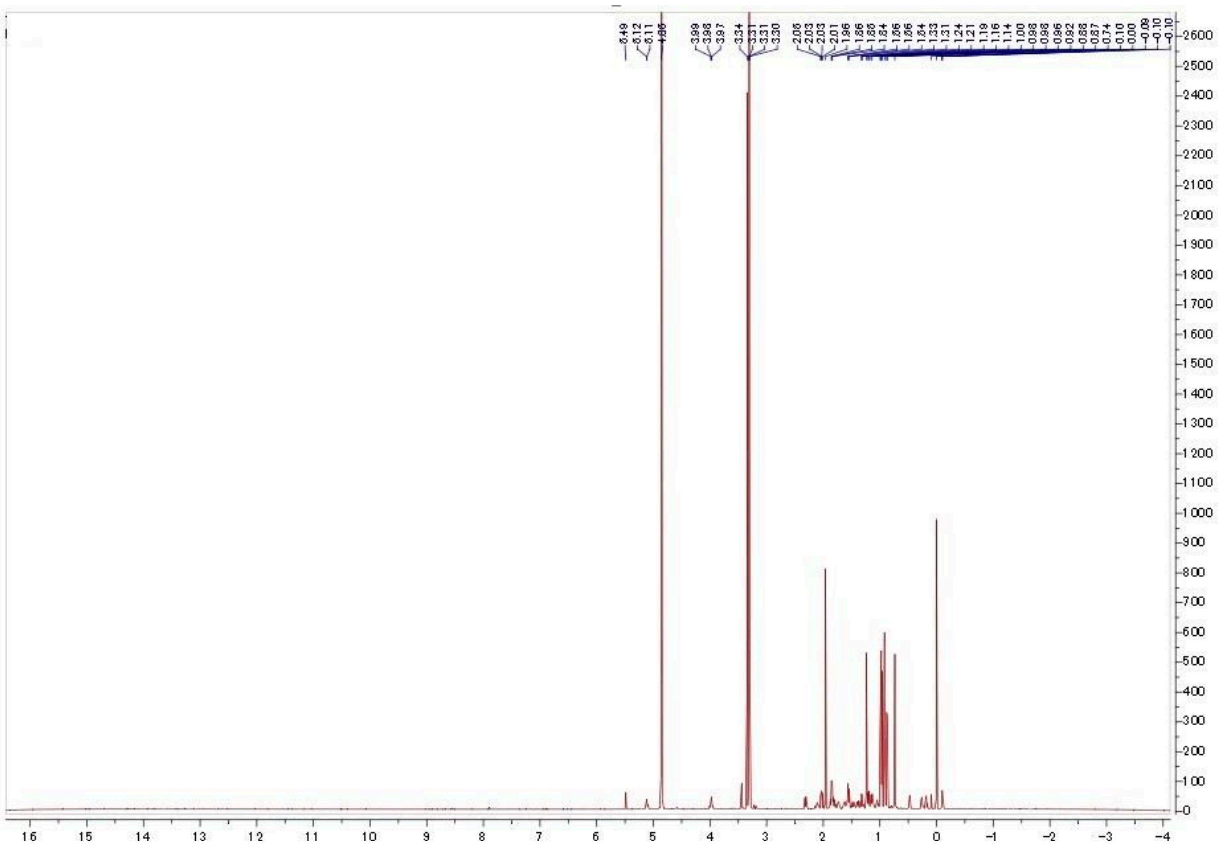

Figure S26. ^{1}H-NMR spectrum of compound **6** (600 MHz, CD3OD).

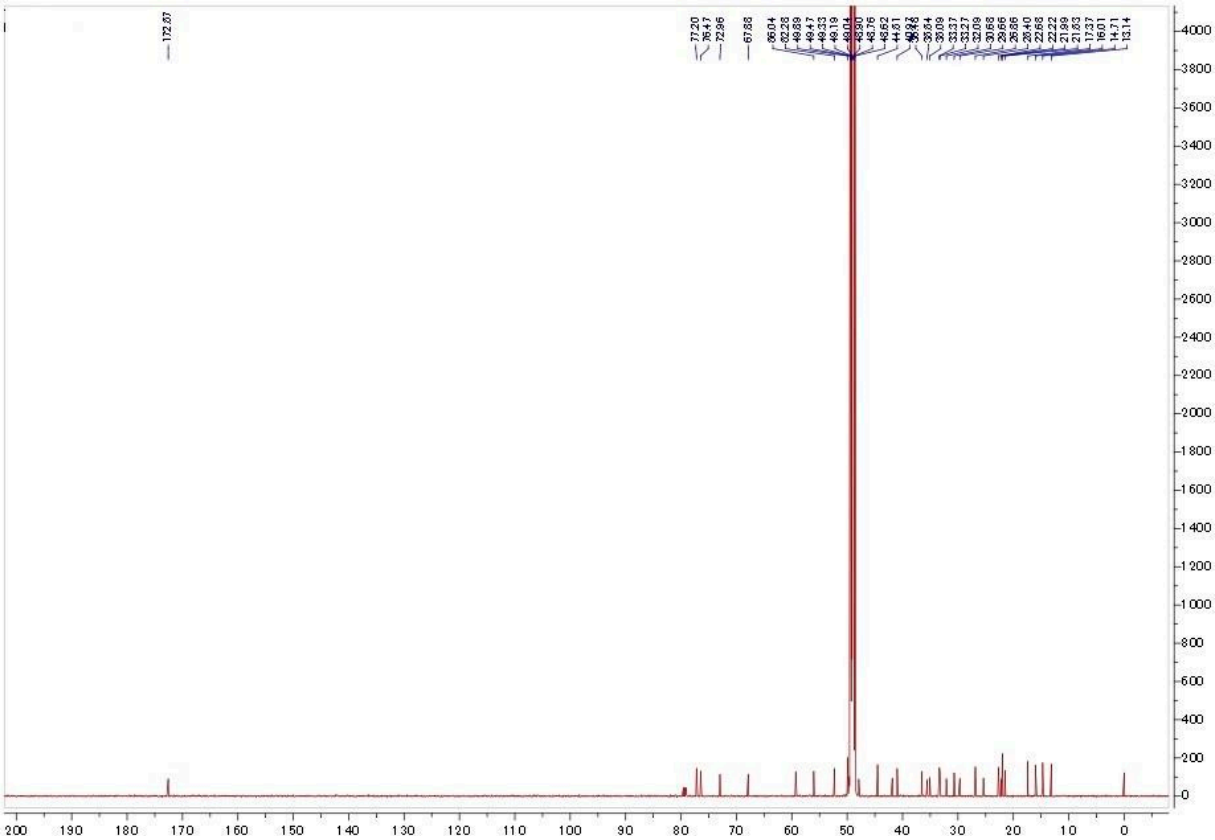

Figure S27. ^{13}C-NMR spectrum of compound **6** (150 MHz, CD3OD).

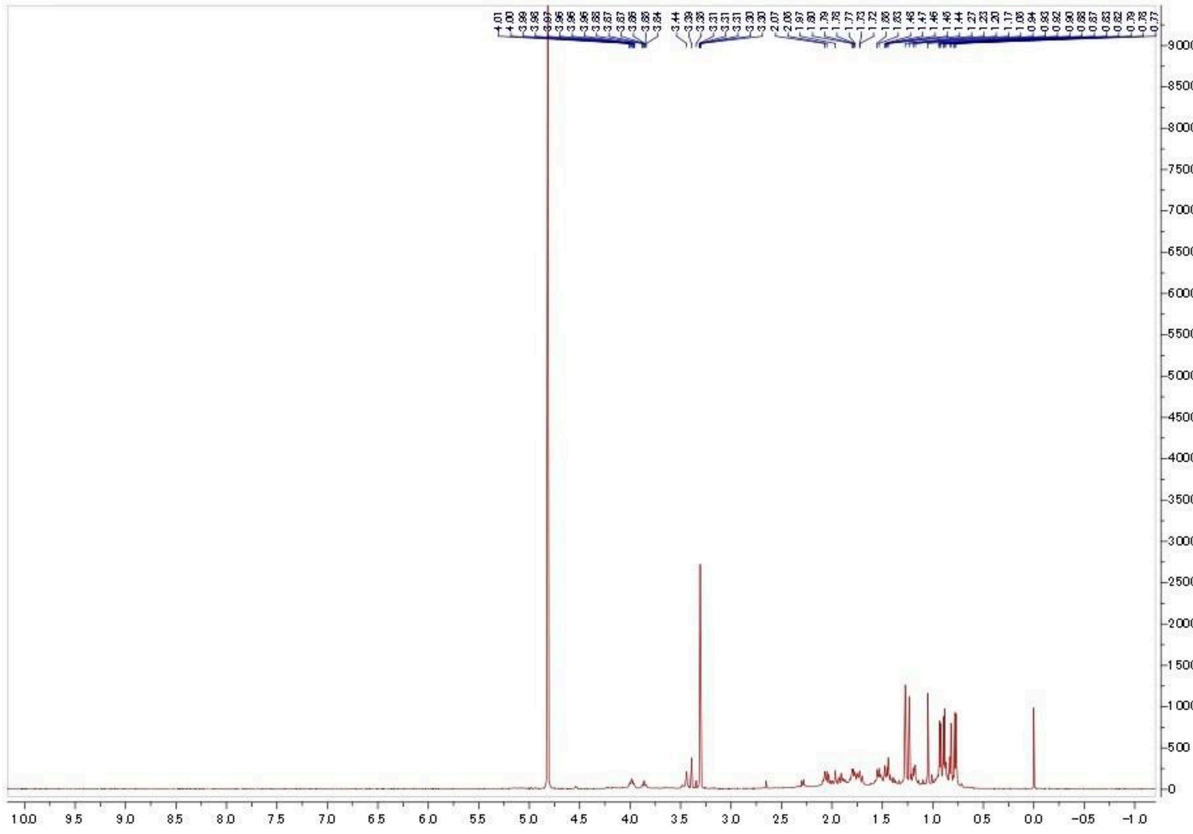

Figure S28. ^{1}H-NMR spectrum of compound **7** (600 MHz, CD3OD).

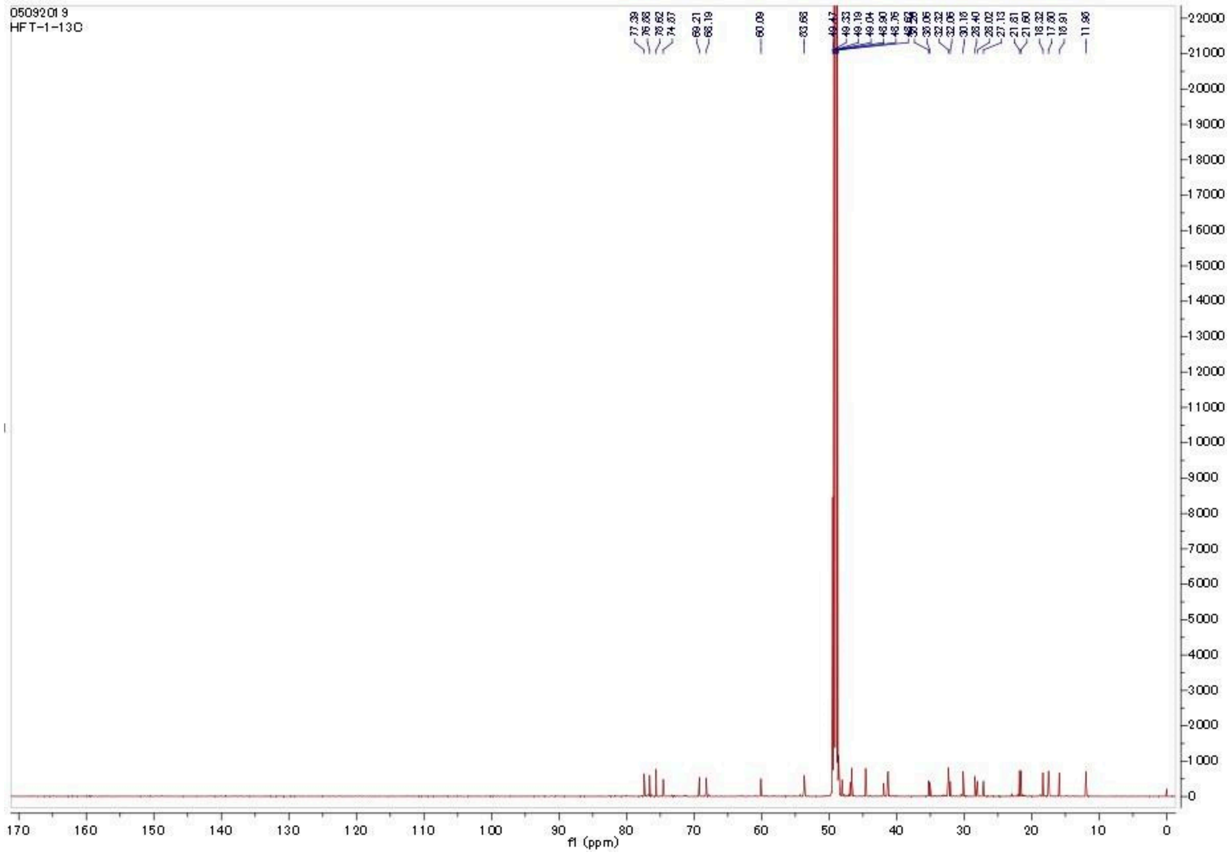

Figure S29. ^{13}C-NMR spectrum of compound **7** (150 MHz, CD3OD).

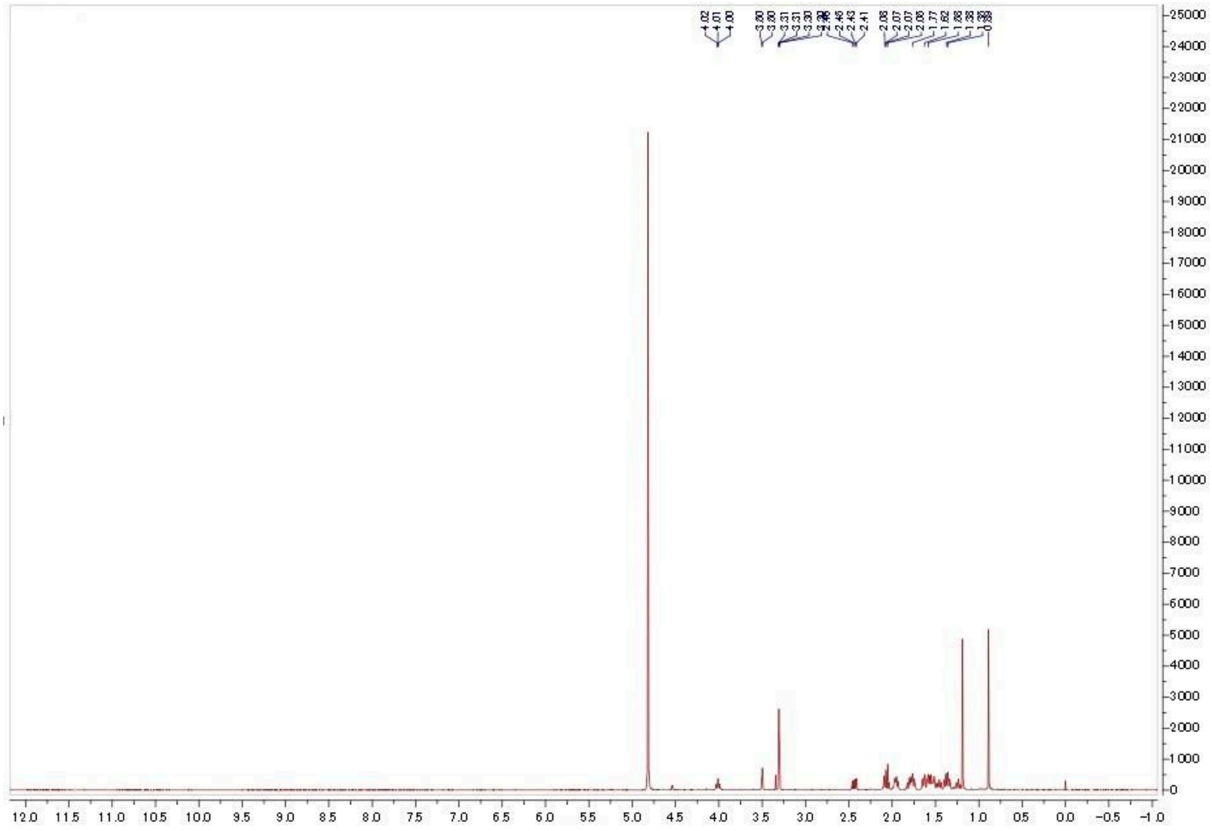

Figure S30. ^{1}H-NMR spectrum of compound **8** (600 MHz, CD3OD).

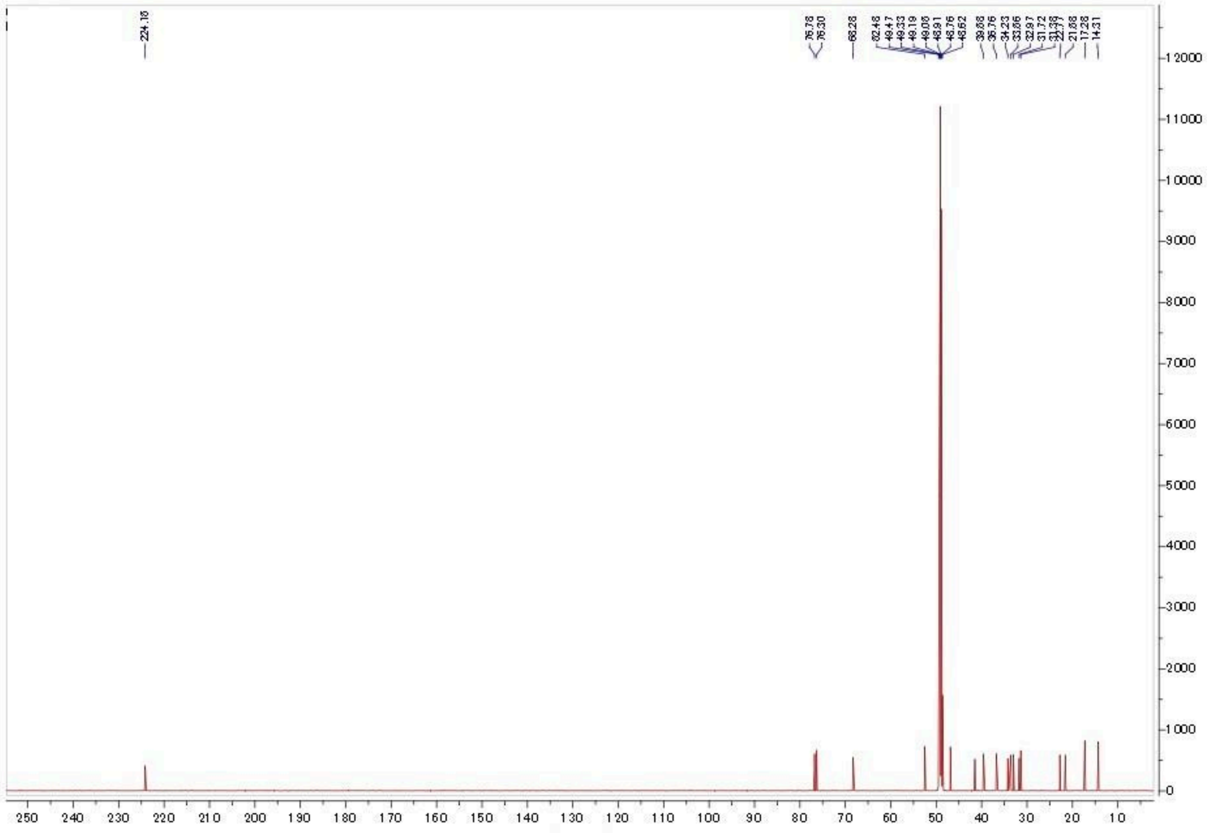

Figure S31. ^{13}C-NMR spectrum of compound **8** (150 MHz, CD_3OD).

Table S4. All SARS CoV-2 related proteins used in this study.

Protein	Name	Function	PDB Code
		**Viral Proteins (Nonstructural proteins)**	
nsp1	-	Host translation inhibitor	7K3N
nsp2	-	Play a role in the modulation of host cell survival signaling pathway byinteracting with host PHB and PHB2	Deposited model in Swiss-Model
nsp3	ADP ribose phosphatase	Activate replicase polyprotein	6W02
nsp4	-	Participates in the assembly of virally-induced cytoplasmic double-mem-brane vesicles necessary for viral replication	Deposited model in Swiss-Model
nsp5 (3CL-PRO)	Main protease	Cleaves the C-terminus of replicase polyprotein at 11 sites	6lu7
nsp6	-	Plays a role in the initial induction of autophagosomes	Deposited model in Swiss-Model
nsp7	-	Plays a role in viral RNA synthesis	6M5I
nsp8	-	Plays a role in viral RNA synthesis	6M5I
nsp9	-	May participate in viral replication by acting as a ssRNA-binding protein	6WC1
nsp10	-	plays an essential role in viral mRNAs cap methylation	6ZPE
nsp11 (PL-PRO)	Papain-like protease	Participate in the activation of the replicase polyprotein	6WX4

nsp12 (Pol; RdRP)	RNA-directed RNA polymer-ase	Responsible for replication and transcription of the viral RNA ge-nome	6M71
nsp13 (Hel)	Helicase	Displaying RNA and DNA duplex-unwinding activities with 5' to 3' po-larity. Activity of helicase is dependent on magnesium	5RL6
nsp14 (ExoN)	Proofreading exoribonucle-ase	An exoribonuclease activity acting on both ssRNA and dsRNA in a 3' to5' direction	Deposited model in Swiss-Model
Nsp15 (NendoU)	Uridylate-spe-cific endoribo-nuclease	Mn(2+)-dependent, uridylate-specific enzyme, which leaves 2'-3'-cyclicphosphates 5' to the cleaved bond.	5S6X
nsp16	2'-*O*-methyl-transferase	Methyltransferase that mediates mRNA cap 2'-O-ribose methyla-tion to the 5'-cap structure of viral mRNAs	6W4H
**Structural proteins**			
S glycoprotein	Spike glyco-pro-tein	Binding to human ACE2 receptor and internalization of the virus into the endosomes of the host cell induces conformational changes in theSpike glycoprotein	6lzg and 6zb5
E-protein	Envelope small membrane pro-tein	Plays a central role in virus morphogenesis and assembly	7k3g
M-protein	Membrane pro-tein	Component of the viral envelope that plays a central role in virus mor-phogenesis and assembly via its interactions with other viral proteins	Deposited model in Swiss-Model
N-protein	Nucleoprotein	Packages the positive strand viral genome RNA into a helical ribo-nucle-ocapsid (RNP) and plays a fundamental role during virion assem-bly through its interactions with the viral genome and membrane protein M	6M3M
**Accessory Proteins**			
ORF3a	ORF3a protein	Forms homotetrameric potassium sensitive ion channels (viroporin) and may modulate virus release	6XDC
ORF6	ORF6 protein	Disrupts cell nuclear import complex formation by tethering karyo-pherin alpha 2 and karyopherin beta 1 to the membrane	Deposited model in Swiss-Model

ORF7a	ORF7a protein	Plays a role as antagonist of host tetherin (BST2), disrupting its antiviraleffect	6w37
ORF7b	ORF7b protein	-	-
ORF8	ORF8 protein	May play a role in host-virus interaction	7JTL
ORF9b	ORF9b protein	Plays a role in the inhibition of host innate immune response by target-ing the mitochondrial-associated adapter MAVS	6Z4U
**Human-derived Proteins**			
ACE2	Angitensin converting enzyme2	Responsible for attachment of SARS CoV	6LZG
Furin	Furin	Activation of S-protein	6EQX
TMPRSS2	Transmembrane protease, serine 2	Activation of S-protein	Deposited model in Swiss-Model
CatL	Cathepsin L	Activation of S-protein	2YJC
NPR1	Neuropilin 1	Facilitates viral entry	3i97
PHB1	Prohibitin sub-unit1	In the mitochondria, together with PHB2, forms large ring complexes (prohibitin complexes) in the inner mitochondrial membrane (IMM) and functions as chaperone protein that stabilizes mitochondrial respiratoryenzymes and maintains mitochondrial integrity	Deposited model in Swiss-Model
PHB2	Prohibitin sub-unit2	In the mitochondria, together with PHB2, forms large ring complexes (prohibitin complexes) in the inner mitochondrial membrane (IMM) and functions as chaperone protein that stabilizes mitochondrial respiratoryenzymes and maintains mitochondrial integrity	Deposited model in Swiss-Model
AAK1	Adaptor Protein 2 Associated Kinase	Participate in viral endocytosis	4WSQ
GAK	Cyclin-G associated kinase	Participate in viral endocytosis	4y8d

Table S5. Docking scores of the isolated compounds (1–8) against the SARS CoV-2 targets expressed in kcal/mol.

Protein	1	2	3	4	5	6	7	8
nsp1	−7.36	−5.23	−4.38	−3.78	−6.98	−3.56	−4.26	−6.36
nsp2	-5.34	-6.31	-6.42	-5.29	-4.27	-3.84	-6.19	-7.36
nsp3	-5.39	-6.22	-5.92	-7.34	-7.88	-7.23	-6.32	-7.13
nsp4	-5.29	-7.44	-4.23	-7.66	-6.95	-7.39	-7.29	-7.48
**nsp5 (M^{pro})**	-4.55	-6.45	-7.73	-8.85	-7.28	-7.42	-8.91	-9.43
nsp6	-6.67	-6.43	-6.94	-5.83	-5.28	-5.29	-5.11	-7.38
nsp7	-7.33	-6.29	-7.27	-6.62	-7.21	-7.73	-7.27	-6.22
nsp8	-5.19	-6.37	-6.44	-7.28	-7.11	-7.37	-6.29	-5.57
nsp9	-7.44	-6.44	-7.16	-6.29	-6.11	-6.38	-6.39	-5.39
nsp10	-5.28	-5.39	-5.88	-6.39	-5.33	-5.22	-7.28	-7.39
nsp11 (PL-PRO)	-6.65	-6.33	-7.98	-6.33	-6.23	-7.44	-5.34	-7.54
nsp12 (Pol; RdRP)	−6.45	−7.44	−6.35	−7.39	−6.45	6.39	−5.44	−6.32
nsp13 (Hel)	-5.29	-6.32	-7.39	-5.55	-7.29	-6.77	-5.28	-5.39
	-5.16	-7.18	-7.42	-7.49	-6.69	-6.99	-7.17	-7.38

nsp14 (ExoN)								
Nsp15 (NendoU)	-5.02	-6.05	-7.12	-5.28	-7.02	-6.5	-5.01	-5.12
nsp16	-4.89	-6.91	-7.15	-7.22	-6.42	-6.72	-6.9	-7.11
S glycoprotein	-6.18	-7.17	-6.08	-7.12	-6.18	6.66	-5.17	-6.05
E-protein	-6.38	-6.06	-7.71	-6.06	-5.96	-7.17	-5.07	-7.27
M-protein	-5.01	-5.12	-5.61	-6.12	-5.06	-4.95	-7.01	-7.12
N-protein	-7.17	-6.17	-6.89	-6.02	-5.84	-6.11	-6.12	-5.12
ORF3a	-4.92	-6.1	-6.17	-7.01	-6.84	-7.1	-6.02	-5.3
ORF6	-7.06	-6.02	-7	-6.35	-6.94	-7.46	-7	-5.95
ORF7a	-6.4	-6.16	-6.67	-5.56	-5.01	-5.02	-4.84	-7.11
ORF7b	-5.02	-7.17	-3.96	-7.39	-6.68	-7.12	-7.02	-7.21
ORF8	-5.12	-5.95	-5.65	-7.07	-7.61	-6.96	-6.05	-6.86
ORF9b	-5.07	-6.04	-6.15	-5.02	-4	-3.57	-5.92	-7.09
ACE2	-7.04	-4.96	-4.11	-3.51	-6.71	-3.29	-3.99	-6.09
Furin	-4.75	-5.78	-6.85	-5.01	-6.75	-6.23	-4.74	-4.85
TMPRSS2	-4.62	-6.64	-6.88	-6.95	-6.15	-6.45	-6.63	-6.84
CatL	-6.9	-5.9	-6.62	-5.75	-5.57	-5.84	-5.85	-4.85
NPR1	-4.85	-5.68	-5.38	-6.8	-7.34	-6.69	-5.78	-6.59
PHB1	-6.77	-4.69	-3.84	-3.24	-6.44	-3.02	-3.72	-5.82
PHB2	-4.75	-5.78	-6.85	-5.01	-6.75	-6.23	-4.74	-4.85
AAK1	-6.42	-6.88	-5.33	-4.16	-6.33	-7.29	-6.97	-5.23
**GAK**	-5.53	-6.87-	-9.26	-8.37	-8.68	-6.44	-7.23	-9.45

1. Docking and Molecular Dynamic Simulation

1.1. Molecular Docking

Auto Dock Vina software was used in all molecular docking experiments [1]. All isolated compounds were docked against the Mpro crystal structure (PDB codes: 7L0D) [2]. The binding site was determined according to the enzyme's co-crystallized ligand. The co-ordinates of the grid box were: x = -12.87; y = 16.3; z = 68.64. The size of the grid box was set to be 15 Å. Exhaustiveness was set to be 24. Ten poses were generated for each docking experiment [3,4]. Docking poses were analyzed and visualized using Pymol software [1].

1.2. Molecular Dynamics Simulation

Desmond v. 2.2 software was used for performing MDS experiments [5-7]. This software applies the OPLS-2005 force field. Protein systems were built using the System Builder option, where the protein structure was checked for any missing hydrogens, the protonation states of the amino acid residues were set (pH = 7.4), and the co-crystalized water molecules were removed. Thereafter, the whole structure was embedded in an orthorhombic box of TIP3P water together with 0.15 M Na^+ and Cl^- ions in 20 Å solvent buffer. Afterward, the prepared systems were energy minimized and equilibrated for 10 ns. For protein-ligand complexes, the top-scoringposes were used as a starting point for simulation. Desmond software automaticallyparameterizes inputted ligands during the system building step according to the OPLS forcefield. For simulations performed by NAMD 2.9.1 [8], the protein structures were built and optimized by using the QwikMD toolkit of the VMD software. The parameters and topologies of the compounds were calculated using the Charmm27 force field with the online software Ligand Reader and Modeler (http://www.charmm-gui. org/?doc=input/ligandrm, accessed on 22 May 2022) [9]. Afterward, the generated parameters and topology files were loaded to VMD to

readily read the protein–ligand complexes without errors and then conduct thesimulation step.

1.3. Absolute binding Free energy calculation

Binding free energy calculations (ΔG) were performed using the free energy perturbation (FEP) method [9]. This method was described in detail in the recent article by Kim and coworkers [9]. Briefly, this method calculates the binding free energy $\Delta G_{binding}$ according to the following equation:

$$\Delta G_{binding} = \Delta G_{Complex} - \Delta G_{Ligand} \quad (1)$$

The value of each ΔG is estimated from a separate simulation using NAMD software. All input files required for simulation by NAMD can be prepared by using the online website Charmm-GUI (https://charmm-gui.org/?doc=input/afes.abinding).Subsequently, we can use these files in NAMD to produce the required simulations using the FEP calculation function in NAMD 2.9.1. The equilibration (5 ns long) was achieved in the NPT ensemble at 300 K and 1 atm (1.01325 bar) with Langevin piston pressure (for "Complex" and "Ligand") in the presence of the TIP3P water model. Then, 10 ns FEP simulations were performed for each compound, and the last 5 ns of the free energy values was measured for the final free energy values [9]. Finally, the generated trajectories were visualized and analyzed using VMD software. It worth noting that Ngo and co-workers in their recent benchmarking study found that the FEP method of determination of ΔG was the most accurate method in predicting M^{Pro} inhibitors [10].

In regard to Molecular Mechanics-Generalized Born Surface Area (MMGBSA) and Molecular Mechanics Poisson-Boltzmann Surface Area (MMPBSA) embedded in the MMPBSA.py module of AMBER18 were utilised to calculate the binding free energy of the docked complex [11]. 100 frames were processed from the trajectories in total, and the system's net energy was estimated using the following equation:

$$\Delta G_{Binding} = \Delta G_{Complex} - \Delta G_{Receptor} - \Delta G_{Inhibitor} \quad (2)$$

Each of the aforementioned terms requires the calculation of multiple energy components, including van der Waals energy, electrostatic energy, internal energy from molecular mechanics, and polar contribution to solvation energy

References

1. Seeliger, D.; de Groot, B.L. Ligand docking and binding site analysis with PyMOL and Autodock/Vina. J. Comput. Aided Mol. Des. 2010, 24, 417–422.
2. Clyde, A., Galanie, S., Kneller, D. W., Ma, H., Babuji, Y., Blaiszik, B., ... & Stevens, R. (2021). High Throughput Virtual Screening and Validation of a SARS-CoV-2 Main Protease Non-Covalent Inhibitor. *bioRxiv.*
3. Sayed, A.M.; Alhadrami, H.A.; El-Gendy, A.O.; Shamikh, Y.I.; Belbahri, L.; Hassan, H.M.; Abdelmohsen, U.R.; Rateb, M.E. Microbial natural products as potential inhibitors of SARS- CoV-2 main protease (Mpro). Microorganisms 2020, 8, 970.
4. Amaro, R.E.; Baudry, J.; Chodera, J.; Demir, Ö .; McCammon, J.A.; Miao, Y.; Smith, J.C. Ensemble docking in drug discovery. Biophys. J. 2018, 114, 2271–2278.
5. Bowers, K.J.; Chow, D.E.; Xu, H.; Dror, R.O.; Eastwood, M.P.; Gregersen, B.A.; Klepeis, J.L.; Kolossvary, I.; Moraes, M.A.; Sacerdoti, F.D.; et al. Scalable algorithms for molecular dynamics simulations on commodity clusters. In Proceedings of the SC'06: Proceedings of the 2006 ACM/IEEE Conference on Supercomputing, Tampa, FL, USA, 11–17 November 2006; IEEE: New York, NY, USA, 2006; p. 43.
6. Release, S. 3: Desmond Molecular Dynamics System, DE Shaw Research, New York, NY, 2017; Maestro-Desmond Interoperability Tools, Schrödinger: New York, NY, USA, 2017.
7. Schrodinger LLC. Maestro, Version 9.0; Schrodinger LLC: New York, NY, USA, 2009.
8. Phillips, J.C.; Braun, R.; Wang, W.; Gumbart, J.; Tajkhorshid, E.; Villa, E.; Chipot, C.; Skeel, R.D.; Kalé, L.; Schulten, K. Scalable molecular dynamics with NAMD. J. Comput. Chem. 2005, 26, 1781– 1802.
9. Kim, S.; Oshima, H.; Zhang, H.; Kern, N.R.; Re, S.; Lee, J.; Rous, B.; Sugita, Y.; Jiang, W.; Im, W. CHARMM-GUI free energy calculator for absolute and relative ligand solvation and binding free energy simulations. J. Chem. Theory Comput. 2020, 16, 7207–7218.

10. Ngo, S.T.; Tam, N.M.; Quan, P.M.; Nguyen, T.H. Benchmark of Popular Free Energy Approaches Revealing the Inhibitors Binding to SARS-CoV-2 Mpro. J. Chem. Inf. Model. 2021,61, 2302–2312.
11. Miller III, B. R., McGee Jr, T. D., Swails, J. M., Homeyer, N., Gohlke, H., & Roitberg, A. E. (2012). MMPBSA. py: an efficient program for end-state free energy calculations. *Journal of chemical theory and computation*, *8*(9), 3314-3321.
